# A Cluster Randomised Trial Introducing Rapid Diagnostic Tests into Registered Drug Shops in Uganda: Impact on Appropriate Treatment of Malaria

**DOI:** 10.1371/journal.pone.0129545

**Published:** 2015-07-22

**Authors:** Anthony K. Mbonye, Pascal Magnussen, Sham Lal, Kristian S. Hansen, Bonnie Cundill, Clare Chandler, Siân E. Clarke

**Affiliations:** 1 Ministry of Health, Box 7272, Kampala, Uganda; 2 School of Public Health, College of Health Sciences, Makerere University, Kampala, Uganda; 3 Centre for Medical Parasitology, Faculty of Health and Medical Sciences, University of Copenhagen, Copenhagen, Denmark; 4 Department of Disease Control, London School of Hygiene and Tropical Medicine, London, United Kingdom; 5 Department of Global Health and Development, London School of Hygiene and Tropical Medicine, London, United Kingdom; 6 Department of Infectious Disease Epidemiology, London School of Hygiene and Tropical Medicine, London, United Kingdom; University of California, San Francisco, UNITED STATES

## Abstract

**Background:**

Inappropriate treatment of malaria is widely reported particularly in areas where there is poor access to health facilities and self-treatment of fevers with anti-malarial drugs bought in shops is the most common form of care-seeking. The main objective of the study was to examine the impact of introducing rapid diagnostic tests for malaria (mRDTs) in registered drug shops in Uganda, with the aim to increase appropriate treatment of malaria with artemisinin-based combination therapy (ACT) in patients seeking treatment for fever in drug shops.

**Methods:**

A cluster-randomized trial of introducing mRDTs in registered drug shops was implemented in 20 geographical clusters of drug shops in Mukono district, central Uganda. Ten clusters were randomly allocated to the intervention (diagnostic confirmation of malaria by mRDT followed by ACT) and ten clusters to the control arm (presumptive treatment of fevers with ACT). Treatment decisions by providers were validated by microscopy on a reference blood slide collected at the time of consultation. The primary outcome was the proportion of febrile patients receiving appropriate treatment with ACT defined as: malaria patients with microscopically-confirmed presence of parasites in a peripheral blood smear receiving ACT or rectal artesunate, and patients with no malaria parasites not given ACT.

**Findings:**

A total of 15,517 eligible patients (8672 intervention and 6845 control) received treatment for fever between January-December 2011. The proportion of febrile patients who received appropriate ACT treatment was 72·9% versus 33·7% in the control arm; a difference of 36·1% (95% CI: 21·3 – 50·9), *p*<0·001. The majority of patients with fever in the intervention arm accepted to purchase an mRDT (97·8%), of whom 58·5% tested mRDT-positive. Drug shop vendors adhered to the mRDT results, reducing over-treatment of malaria by 72·6% (95% CI: 46·7– 98·4), *p*<0·001) compared to drug shop vendors using presumptive diagnosis (control arm).

**Conclusion:**

Diagnostic testing with mRDTs compared to presumptive treatment of fevers implemented in registered drug shops substantially improved appropriate treatment of malaria with ACT.

**Trial Registration:**

ClinicalTrials.gov NCT01194557.

## Introduction

In the past decade, most countries in sub-Saharan Africa, including Uganda, have changed the first-line anti-malarial treatment to artemisinin-based combination therapy (ACT), [1–3]. Yet, effective treatment is hampered by inappropriate treatment of malaria, arising either from missed diagnoses or from patients without malaria being over-treated with anti-malarial drugs. Inappropriate treatment of malaria is widely reported from many countries and occurs in both public health and private retail sectors [4–13]. Where a confirmatory laboratory diagnosis is unavailable, the practice of treating all fevers as malaria results in significant over-treatment, and can lead to non-malaria febrile illnesses remaining untreated, with associated risks for the patient [14–16]. The higher costs of ACT, together with increasing recognition of risk of under-diagnosis of non-malarial fevers, have contributed to the reconsideration of presumptive treatment of malaria. Overuse of ACTs as a result of overtreatment in the private sector is an additional concern where subsidies are used to improve the affordability of quality ACTs to patients, through mechanisms such as the Affordable Medicines Facility—malaria (AMFm) [17]. Rapid diagnostic tests (mRDTs) provide a simple means of confirming malaria diagnosis in remote areas lacking microscopes, infrastructure and qualified health staff. Rapid diagnostic tests are affordable, quick, accurate and relatively easy to perform with minimal training [16, 18–20]. Accordingly, WHO recommends universal access to parasitological diagnosis, encompassing all treatment providers, including the private sector [21].

Although there has been an attempt to roll-out of mRDTs in public health facilities, the introduction and use of mRDTs in the private sector has been much slower. In 2010, Uganda national policy recommended introduction of mRDTs at all levels to improve malaria case management through accurate parasitological diagnosis [22]. Nonetheless, baseline surveys in Mukono district in 2010 confirmed the lack of diagnostic testing in registered drug shops [23]. In Uganda, drug shops play an important role in provision of healthcare with up to 80% of malaria cases treated by these providers and an estimated 50% of all anti-malarial drugs in Uganda are distributed through drug shops [24]. Yet in a recent study, less than 10% of febrile children treated at drug shops received appropriate treatment for malaria [25]. There thus remains an urgent need to improve in diagnosis and treatment practices amongst drug retailers in Uganda and many other African states, particularly in areas where there is poor access to formal health facilities and self-treatment of fevers with anti-malarial drugs bought in shops is the most common form of care-seeking [6–11].

In light of the strong need to improve case management of malaria in the private sector, a cluster randomized trial was designed with the main objective of examining the impact of introducing mRDTs into registered drug shops in Uganda, to encourage rational drug use in case management of malaria. The specific objective was to evaluate the impact of availability of mRDTs in drug shops on appropriate anti-malarial drug prescription, and referral to the formal health sector. It was hypothesised that use of mRDTs in the private sector would increase the proportion of febrile patients receiving appropriate malaria treatment with ACT.

## Methods

### Trial design and study setting

A cluster-randomized controlled trial of mRDT use in registered drug shops was conducted to compare the proportion of patients receiving appropriate treatment with ACT under two diagnostic scenarios: (i) drug shops trained to perform mRDTs to inform malaria case management (intervention arm), and (ii) current practice of presumptive clinical diagnosis and treatment of fever (control arm). In Uganda, registered drug shops occupy an officially recognised role in the Ugandan health system, required to meet minimum standards for registration, including ownership and operation of the business by a qualified health worker and premises suitable for storage of drugs; they are subject to periodic inspection by the District Assistant Drug Inspector (DADI), with a licence renewable annually for a fee [26]. Registered drug shops are licensed to sell non-prescription drugs, including anti-malarial drugs but not antibiotics or injections. The trial was conducted in Mukono district, Central Uganda, an area of year-round malaria transmission. In this region, the prevalence of fever among children aged <5years in 2011 was 42% [27]. The majority of the population, 88%, lives in rural areas, predominantly comprised of subsistence farmers of the Baganda ethnic tribe. The district was chosen because it is highly endemic for malaria, and characteristic of other parts of rural Uganda.

#### Randomization

A cluster was defined as a natural grouping of one or more registered drug shops, within a single parish boundary (administrative area). It could also include drug shops in a neighbouring parish where the distance between drug shops was <1km. To be eligible for inclusion in the trial, a cluster needed to include at least one drug shop registered with the district drug inspector or National Drug Authority; and to be located in a parish containing a health centre II (lowest public health facility where early treatment is sought); and more than 200 households to ensure a sufficient number of patients visiting the drug shops. It was therefore possible for a cluster to have more than one drug shop.

Drug shops not registered with the district, or whose licence had lapsed, were not eligible to participate. Drug shops were often clustered in trading centres with other shops. Thus study clusters included both drug shops located in small trading posts, typically located at crossroads in rural areas, and those located in more developed commercial sites in peri-urban areas. Randomization of clusters was carried out after drug shops had consented to be included into the study. It was not possible to blind participants from the intervention since drug shops with mRDT had posters identifying them.

A baseline survey was carried out in registered drug shops that met the eligibility criteria and is reported elsewhere [23], in brief, the study found that most drug shop staff were female, with the majority having attained secondary level education, and approximately 50% were qualified health workers [23]. Nonetheless, few had previously received any prior specific training on management of malaria or use of an mRDT; and knowledge on first-line treatment for malaria was low. All drug shops reported being open at least five days a week, with the majority (68%) open every day. Opening hours were long, with many shops open up to 10pm at night. Typically, drug shops reported seeing around 10 febrile patients each day (range 2–50 patients).

Allocation of clusters to study arms was performed using a process of restricted randomisation to balance cluster-level factors likely to be associated with the primary outcome [28, 29], using cluster-level data collected at baseline [23]. Balance was defined in terms of covariate-specific thresholds defined as the maximum difference allowed for each of the following characteristics between the study arms: rural-urban classification, number of drug shops per arm, baseline estimate of the primary outcome, and prior training on diagnosis and treatment of malaria. The allocation of clusters was performed as follows:

- Approximate balance on location. Allocations only included if each arm had at least 1 rural cluster (corresponding to a 1:2 or 2:1 split between the arms), at least 10 urban clusters (corresponding to a 5:6 or 6:5 split) and exactly 3 urban/rural clusters per arm.
- Balance to within 3 on the number of drug shops per arm
- Balance to within 10% on the baseline estimate of the primary outcome. Specifically, if the risk ratio was greater than 1.1 or less than 0.9 that allocation was excluded.
- Balance to within 15% on the three training covariates: proportion of DSVs with training in the management of malaria, proportion of DSVs knowing 1^st^ line treatment for malaria, and proportion of DSVs knowing what an mRDT was for.

There were 184,756 ways of allocating half the clusters to the control arm and half to the intervention arm. A compute program (R statistical environment) enumerated each of these allocations and tested whether each of these satisfied all of the balance criteria (described above). A total of 2,713 allocations satisfied the criteria and one was randomly chosen. The validity of the restricted randomisation was assessed by producing a matrix of the probability that each pair of clusters is allocated to the same study arm; which did not indicate any potential cause for concern in the randomisation. In summary, 10 clusters of drug shops were randomly allocated to the intervention arm and 10 clusters to the control arm.

### Description of the Intervention and Control

**Fig 1:**
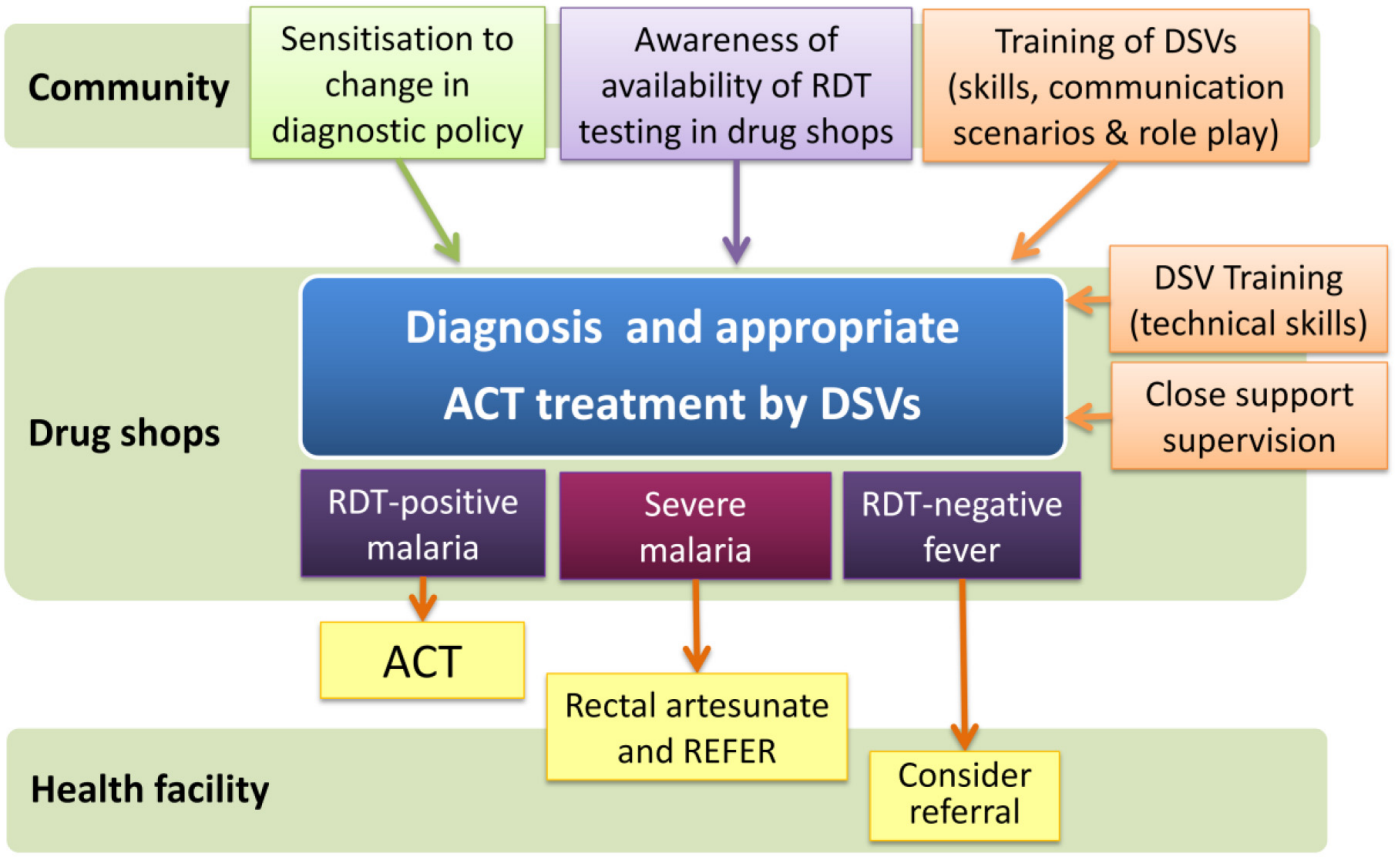
Schematic diagram of the intervention design and treatment outcomes.

The design of the intervention was informed by formative research undertaken between August 2010 to September 2010; including focus group discussions with community members, health workers and drug shop vendors, a baseline survey of registered drug shops in the study area, exit interviews with clients, and a willingness-to-pay study to inform pricing. Findings from the formative research, intervention design and trial protocol are published in full elsewhere [30–33]. In brief, the intervention consisted of providing participatory drug shop vendor training based on a trainer’s manual and accompanying set of pictorial job aids on malaria case management (available from www.actconsortium.org/RDTdrugshops); and provision of subsidised commodities (mRDTs and ACTs) to drug shops (Fig 1). Training and subsidized commodities were provided to both arms to ensure that the only difference between arms was in the method of diagnosis, and that any difference in treatment practices could not be due to a difference in provider knowledge or access to subsidized goods.

#### Training of drug shops

All registered drug shops located in eligible clusters and staff were invited to attend a training workshop in malaria diagnosis and treatment in October 2010. Drug shop vendors (DSVs) in the control arm were trained in presumptive diagnosis of malaria; and the training support materials and procedures were identical in every respect except for the method of diagnosis to be used. DSVs in both arms were trained in the signs and symptoms of malaria, signs for referral, administration of artemether-lumefantrine for uncomplicated malaria and rectal artesunate suppositories for severe malaria, communication skills, record keeping and stock management. Both arms were also trained on the completion of record forms and study procedures, including preparation of blood slides for reference microscopy. The intervention arm received additional one-day training to cover the rationale for diagnostic testing in febrile patients, performing an mRDT and interpretation of the test result based on training materials from WHO [34]. Training encouraged interactive discussion and reflective practice, supported by role play to practise skills necessary to obtain clinical history, explain outcome of an mRDT test, treatment given, and referral advice. DSVs were advised to consider giving referral advice to clients who presented with signs of severe disease or conditions they were unable to manage. Training workshops lasted 4 days for DSVs in the mRDTs intervention arm and 3 days for DSVs in the control arm and were conducted by a team composed of the District health educator, and senior project staff. Participants were assessed at the beginning and end of the training; certificates with a Ministry of Health logo were issued to DSVs who had successfully completed the course in both arms, to build trust amongst community and health staff and presented in the presence of local community leaders and district health officials on the final day of training.

After training, artemether-lumefantrine tablets and rectal artesunate were supplied to all drug shops in both arms, requisitioned free of charge from the project. DSVs were asked to sell ACT and mRDT at agreed recommended prices (an mRDT was sold at US$0.2 and a course of ACT at US$0.4–1.2 depending on age). These prices were informed by a prior willingness-to-pay study among drug shop customers [32]. DSVs in the intervention arm were also provided with roadside signage advertising the availability of malaria diagnosis. For the first two months of implementation (November-December 2010), DSVs in both arms received additional close support supervision with weekly site visits by a dedicated field supervisor to assist adoption of the new procedures into everyday practice and to promote accurate and complete record keeping. Pictorial job aids and detailed standard operating procedures (SOPs) for the DSVs and supervisors were provided for quality assurance in both arms to ensure comparability. DSVS were provided with the supervisor’s telephone number, to call whenever they encountered any difficulties, for timely support. After two months, support supervision was scaled down to DSVs who occasionally experienced problems and asked for help.

### Supporting interventions

Recognising that drug shop vendors may need to broker the change in diagnostic practice with patients, the DSV training included practise in communication skills necessary to explain the rationale for diagnostic testing, result of the mRDT test and treatments given.

In addition, community sensitization on diagnostic testing for malaria was carried out throughout the study area prior to the trial (including communities in both arms) by Village Health Teams (VHTs), trained by the research team and district health authorities. VHTs spoke at community meetings, church gatherings, sometimes utilising local loudspeaker systems, and distributed leaflets. The key messages were: the new policy change on malaria diagnosis and treatment (that not all fevers are malaria and hence a laboratory diagnosis was advisable before treatment with ACT); that a quick malaria test (mRDT) could test for malaria, and that these tests were available locally from government health facilities and trained drug shop vendors.

### Evaluation of Outcomes

Data on all consultations by patients presenting at the drug shop with a fever or history of fever were captured prospectively; symptom history, diagnosis and any treatment received were routinely recorded by DSVs in a register provided for this purpose. The treatment decisions made by providers over a 12-month period between January-December 2011, after the end of the close support supervision, were validated by light microscopy on a reference blood slide collected from the patient by the DSV at the time of consultation, and appropriate targeting of ACTs compared between arms. The primary endpoint assessed in both arms was the proportion of febrile patients with malaria receiving appropriate treatment with a 1^st^-line anti-malarial, a composite outcome defined as: malaria patients with microscopically-confirmed presence of parasites in a peripheral blood smear receiving either artemether-lumefantrine or rectal artesunate (depending on severity of symptoms), and febrile patients with no malaria parasites (slide-negative) who do not purchase an ACT (denominator: all consultations for fever or history of fever). A co-primary outcome was over-prescription defined as the proportion of patients not parasite-positive (slide negative) who receive inappropriate ACT treatment from a drug shop (denominator: patients with a negative research slide). A secondary outcome on prompt effective treatment, defined as the proportion of febrile patients seen at a registered drug shop who receive appropriately targeted ACT treatment within 24 hours of onset of malaria symptoms, was included in order to evaluate the effect of the intervention on access to prompt, effective treatment. For example, if mRDT testing were to substantially delay treatment seeking (for example due to cost of test) this potentially diminishes the public health benefit of the intervention.

Drug shop vendors seeing febrile patients with severe malaria receiving rectal artesunate were trained to refer them to nearest health facility, and also to consider referral for those with mRDT negative fevers. DSVs recorded the symptoms and identifying information of patients referred to formal health facilities on a carbon copy form, gave one copy to the patient, retained one for their records and sent the another to the project office; health units in the study area were requested to complete and retain referral forms from patients referred by DSVs to facilitate data linkage. A qualitative evaluation was undertaken to explore the intended and unintended effects of the intervention and referral process for patients, drug shop vendors and other health care providers [35]. In addition, a random sample of 500 patients was visited at home on the fourth day after the drug shop consultations. The aim of the follow up was to collect data on household costs associated with treatment, and assess price adherence by DSVs; patient adherence to 3-day ACT regimen and/or referral advice; and conduct active surveillance for adverse events [36]. Field assistants visited DSVs according to a randomised schedule to identify clients with fever seen in the previous 4 days. They obtained addresses recorded by DSVs and telephone numbers to locate selected homes. To minimise the risk that the evaluation might influence provider behaviour, patient follow-up interviews and focus group discussions were not conducted within the first 12-months of implementation.

#### Sample size calculation

Sample size calculations were based on the primary outcome of appropriate treatment and informed by results observed from our baseline survey [23]. Assuming a prevalence of appropriate treatment of 32% in the control arm, a between-cluster coefficient of variation (*k*) of 0.15, and 10 clusters per arm, a sample size of 319 febrile patients per cluster would allow us to detect an absolute increase of 8 percentage points (to 40% in the intervention arm) with 80% power at 5% significance level. During the course of the trial calculations were revisited to account for unequal number of clusters per arm due to a cluster drop-out. The revised sample size per cluster was 469 febrile patients. The sample size for household interviews was determined with an objective to detect a decrease of at least 30% of mean household cost of treatment seeking in the intervention arm from a mean cost of 3,500 Uganda shilling in the control arm; informed by previous research [32]. Assuming k = 0.25, power of 80%, a significance level of 5% and 10 clusters per arm, this required 250 interviews per arm.

#### Laboratory methods

A finger-prick blood sample was collected by the DSV (in both arms) after eliciting verbal consent from the client at the time of consultation; and a thick blood film for microscopy prepared and an mRDT (First Response; intervention arm only) performed. The mRDTs passed independent quality-assurance batch testing by WHO-FIND, Institut Pasteur, Cambodia. Blood smears from both arms were stored at drugs shops in slide boxes provided by the project, submitted to the project office weekly, together with patient record forms and used mRDTs. Used mRDTs were re-read at the project office and used to validate accuracy of RDT interpretation and reporting by DSVs. Slides were haemolysed and stained with 10% Giemsa for 10–15 minutes. Malaria parasites were counted against 200 leukocytes and expressed as number of parasites per μl of blood assuming a standard leukocyte count of 8,000/μl of blood. A blood smear was declared negative after examining 100 high power fields with no parasites seen. Laboratory technicians were trained to diploma level and were experienced with at least 5 years of professional experience. All reference slides were subjected to two independent readings by light microscopy and discrepant results based on whether the slide was positive or negative were resolved by a third technician blinded to the previous results.

#### Statistical methods

A modified intention-to-treat cluster level analysis was undertaken using methods appropriate for a small number of clusters per arm [28, 37]. A measure of the outcome of interest was estimated for each cluster. These cluster-level summaries were analysed using an unpaired t-test to provide risk differences, 95% confidence intervals (CI), and to test the null hypothesis of no intervention effect. Adjustment for covariates was conducted using a two-stage procedure. Firstly, the probability of the outcome of interest was fitted from a regression model including the covariates of interest but excluding study arm. Expected values from this model were computed and compared with observed values, to provide a difference-residual for each cluster. At the second stage these cluster-level summaries were analysed using an unpaired t-test to give adjusted risk difference and 95% confidence interval.

Data was double entered and verified using Microsoft Access 2007 (Microsoft Inc., Redmond, Washington) and analysed using STATA version 11.0 (STATA Corporation, College Station, Texas). The analytical plan was approved by an independent data safety and monitoring board (DSMB) prior to analysis.

### Ethical considerations

**Fig 2:**
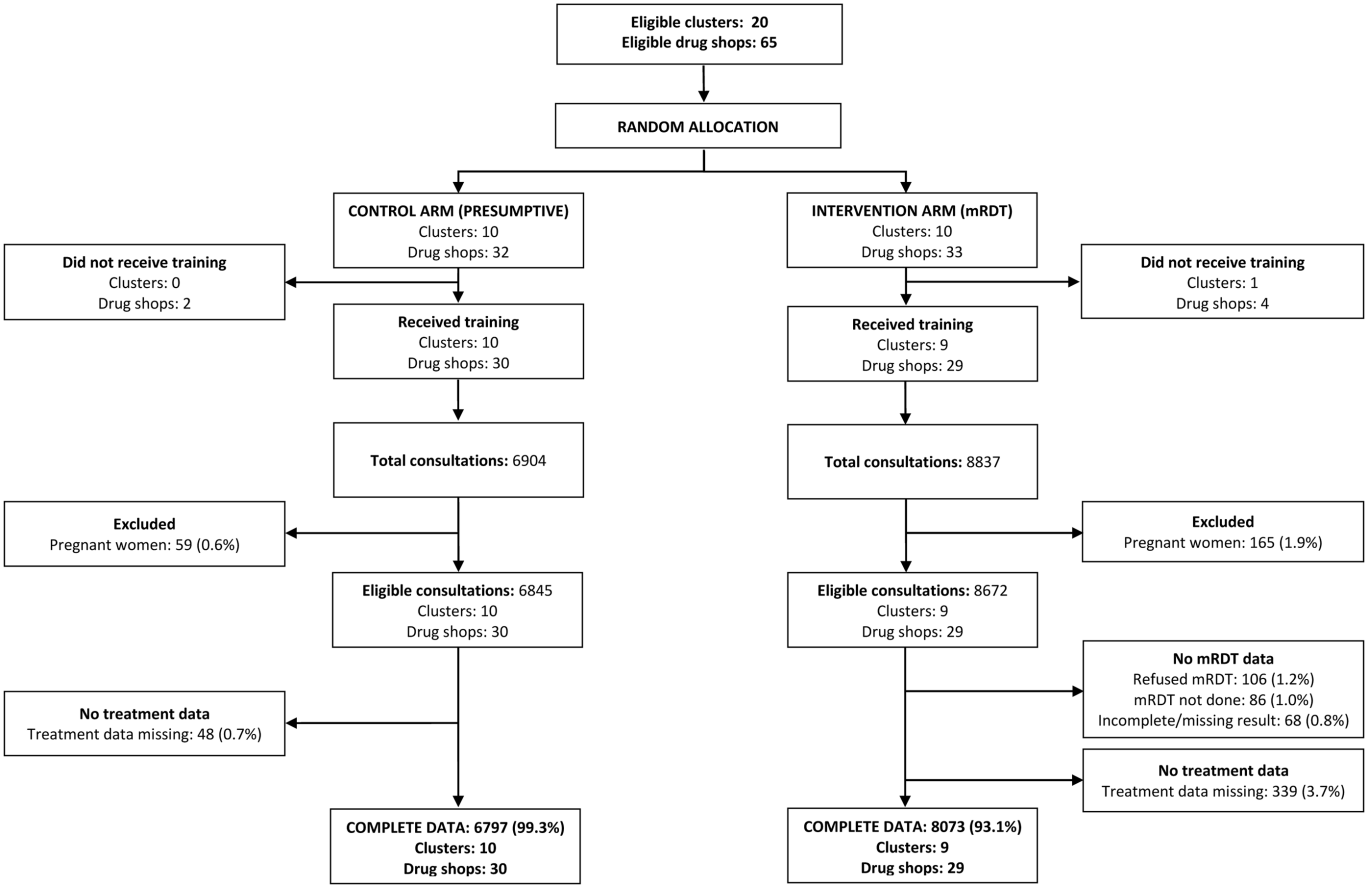
Trial profile appropriate treatment.

Ethical approval for the research was granted by review boards at the Uganda National Council of Science and Technology and London School of Hygiene and Tropical Medicine. Written informed consent was obtained from drug shop vendors to participate in the trial, and from the patient (or caregiver) prior to household interviews. Verbal consent was sought from patients at the time of drug shop consultation for an mRDT and/or research blood slide. Refusals for blood samples were documented, and summarised in Fig 2

## Results

A total of 65 registered drug shops (33 intervention arm and 32 in the control arm) were eligible and consented to participate in the trial and baseline survey; there were no refusals (Fig 2). Nevertheless, six drug shops failed to attend the training because they thought the intervention would impact detrimentally on their business. Since other drug shops in the same clusters did attend the training this only resulted in the loss of one complete cluster, in the intervention arm. The trial was implemented in the 59 drug shops that had attended the training (29 intervention, 30 control). Nine drug shops dropped out of the study during the course of the trial (2 intervention, 7 control) due to closure of the outlets. Consultation data until time of drop out were included in analysis.

The 59 drug shops that participated in the trial were located either in urban sites or established rural trading centres; staff were mainly female, of whom over 50% were qualified health workers (state enrolled nurse or above). The baseline surveys with drug shop vendors revealed that the majority of vendors had received no prior training on malaria case management, had little knowledge of ACT as the first-line anti-malarial drug or what an mRDT was used for [23]. These characteristics were generally balanced between arms; except that DSVs in the intervention arm were less qualified compared with control and fewer had received prior training in malaria case management (Table 1). Nevertheless, prescription practices assessed through patient exit interviews at baseline were similar between the two arms. Malaria microscopy showed less than a third of patients seeking treatment for fever from a drug shop to be infected with malaria parasites. Slide positivity amongst drug clients at baseline was similar, 26.3% and 28.7% in the control and intervention arms respectively.

**Table 1:** Characteristics of drug shop staff and prescription practice at baseline by study arm^1^.

	Control arm	Intervention arm
	Frequency (%)	Frequency (%)
**Characteristics of drug shops**:		
Number of clusters	n = 10	n = 9
Number of drug shop vendors interviewed	n = 30	n = 29
*Location of drug shop*:		
Urban areas/major trading centres	24 (80·0)	23 (79·3)
Rural areas/minor trading centres	6 (20·0)	6 (20·7)
**Characteristics of drug shop vendors**:		
*Gender*:		
Male	6 (20·0)	6 (20·7)
Female	24 (80·0)	23 (79·3)
*Education*:		
Secondary	29 (96·7)	28 (96·6)
Tertiary	1 (3·3)	1 (3·4)
*Level of professional training* ^*2*^:		
High (enrolled nurse, midwife, clinical officer)	17 (56·7)	11 (37·9)
Low (auxiliary nurse, nursing aide)	13 (43·3)	18(62·1)
*Ever trained in malaria case management*:		
Artemisinin combination therapy (ACT)	10	6
Integrated management of childhood illness (IMCI)	7	5
Rapid diagnostic tests (mRDT)	2	0
Microscopy	0	0
None of the above	16 (53.3)	19 (65.5)
*Knowledge of ACT as first-line treatment*:		
Yes	13 (43·3)	12 (41·4)
No	17 (56·3)	17 (58·6)
*Knowledge of what an RDT is used for*:		
Yes	8 (26·7)	10 (34·5)
No	22 (73·3)	19 (65·5)
**Prescription practice prior to intervention**:		
Number of patient exit interviews	n = 261	n = 232
Proportion of patient presented within 24hrs (95%CI)	37·5 (31·6–43·4)	32·8 (26·7–38·8)
Number of patients with a blood slide positive for *P*.*falciparum* (%)	73 (26·3)	72 (28·7)
Over-treatment: proportion of blood slide negative patients receiving ACT (95%CI)	18·9 (9·5–28·2)	19·0 (9·5–28·5)
Proportion of febrile patients receiving appropriately targeted treatment of malaria with ACT (95%CI)	34·1 (26·9–41·4)	32·2 (19·4–45·1)

^1^ Data were collected during interviews with drug shop vendors and patient exit interviews during baseline surveys in May-September 2010 prior to training. One drug shop vendor was interviewed in each shop.

^2^ High level of training included enrolled nurse 20; midwife 5; clinical officer 3; other nurse 2. Low level of training included auxiliary nurse 14; nursing aide 21.

**Fig 3:**
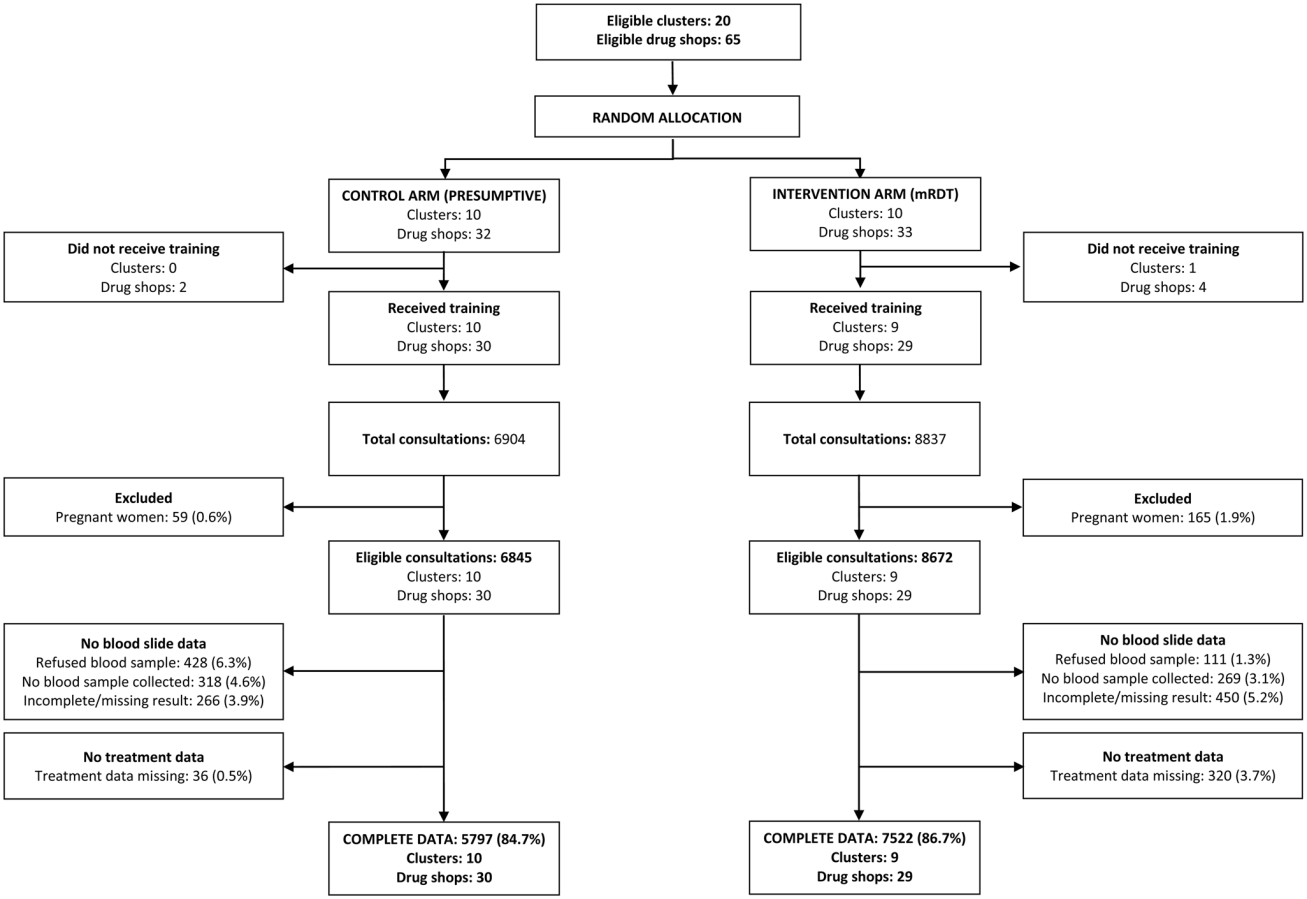
Trial profile provider adherence to mRDTs.

Over the 12-month period January-December 2011, following the end of close-support supervision, a total of 15,741 febrile patients sought treatment from a drug shop enrolled in the trial; 8,837 intervention, 6,904 control. Drug shop vendors had been trained to perform a mRDT test and refer mRDT-positive pregnant women to a health facility for assessment of gestational age prior to ACT treatment. Consultations by pregnant women were therefore excluded from analysis [165 (2%) intervention, 59 (1%) control arm]. The effect of mRDT use on decisions made by DSVs to prescribe ACTs was evaluated in 8,073 non-pregnant clients who had complete data on mRDT result and treatment received in the intervention arm, and in 6,797 non-pregnant clients who had data on treatment received in the control arm; comprising 99·3% and 93·1% of eligible consultations respectively (Fig 2). Appropriate targeting of ACT treatments was evaluated in non-pregnant clients who had complete data on both research slide microscopy and treatment received (7,522 intervention, 5,797control); comprising 86·7% and 84·7% of eligible consultations respectively (Fig 3). Blood slide data were missing or incomplete for 9·6% of eligible consultations in the intervention arm, and treatment data was missing for a further 320 clients (3·7%). Some clients refused to provide a blood sample in the control arm and blood slide data were thus missing or incomplete for 14·7% of eligible consultations; and treatment data missing from a further 36 (0·5%).

Patient characteristics differed slightly between the two arms: in age, time since of onset of symptoms, and reported net use (Table 2).

**Table 2:** Characteristics of patients seen at drug shops during the trial (Jan–Dec 2011).

		Control arm	Intervention arm
		Frequency (%)	Frequency (%)
**Patients with complete data on mRDT adherence** ^**1**^		n = 6797	n = 8073
Age of patient	<5 years	2306 (34.3)	3098 (38.7)
	5–15 years	2201 (32.7)	2082 (26.0)
	16–60 years	2098 (31.2)	2653 (33.1)
	60+ years	124 (1.8)	172 (2.2)
Sex of patient	Male	3280 (48.3)	3908 (48.5)
	Female	3513 (51.7)	4152 (51.5)
Time since onset of symptoms	Within 24 hours	5508 (68.9)	5669 (84.5)
Slept under a mosquito net the previous night	Yes	3585 (53.0)	5242 (65.3)
	No	3178 (47.0)	2781 (34.7)
		Frequency (%)	Frequency (%)
**Patients with complete data on appropriately targeted treatment (primary endpoint)** ^**2**^		n = 5797	n = 7522
Age of patient	<5 years	2062 (35·9)	2860 (38·4)
	5–15 years	1900 (33·1)	1959 (26·3)
	16–60 years	1682 (29·3)	2475 (33·2)
	60+ years	100 (1·7)	163 (2·2)
Sex of patient	Male	2792 (48·2)	3632 (48·4)
	Female	3001 (51·8)	3878 (51·6)
Time since onset of symptoms	Within 24 hours	4807 (84·0)	5162 (69.3)
Slept under a mosquito net the previous night	Yes	3046 (52·8)	4904 (65·6)
	No	2721 (47·2)	2568 (34·4)

^1^ Data missing for: age 136 (68 intervention, 68 control); gender: 17 (13 intervention, 4 control); onset of symptoms: 169 (81 intervention, 88 control); net use: 84 (50 intervention, 34 control)

^2^ Data missing for: age 118 (65 intervention, 53 control); gender: 16 (12 intervention, 4 control); onset of symptoms: 143 (71 intervention, 72 control); net use: 80 (50 intervention, 30 control)

### Provider adherence to mRDT test results and effect on ACT prescription by drug shop vendors

The majority of patients in the intervention arm (8,480/8,672; 97·8%) accepted to purchase an mRDT of whom 58·5% were reported to be mRDT positive. Used mRDTs were routinely collected and re-read by the research team; 95% of these readings were concordant with the mRDT result recorded by the DSV (Kappa statistic 0·89, p<0·001). There was high adherence by DSVs to the results of the mRDT testing, with only 49 (1.5%) of mRDT negative patients receiving artemether-lumefantrine or rectal artesunate pre-referral treatment (hereafter referred to collectively as ACT treatment) (Fig 3, Table 3). Anti-malarial prescription overall was markedly reduced by use of mRDT diagnostic testing; only 62·5% of clients in the mRDT intervention arm received an ACT, compared with almost 100% of clients treated with ACTs in the presumptive diagnosis arm; equating to a risk difference of -37·6% (CI -51·8 to -23·4), *p*<0·001, adjusted for the cluster randomised design and imbalances between arms at baseline (Table 3).

**Table 3:** Diagnosis and treatment of malaria at registered drug shops: Effect of mRDTs on ACT treatments prescribed by drug shop vendors.

	**Control arm**	**Intervention arm**	**Adherence in Intervention arm according to mRDT result**			
	Frequency (%)	Frequency (%)	**mRDT negative**		**mRDT positive**	
Clients with complete data on trial outcome	n = 6797	n = 8073	n = 3166		n = 4907	
Prescribed artmether-lumefantrine	6732 (99.0)	4856 (60.2)	42 (1.3)		4814 (98.1)	
Prescribed rectal artesunate	49 (0.7)	51 (0.6)	7 (0.2)		44 (0.9)	
Did not receive AL or rectal artesunate	16 (0.2)	3166 (39.2)	3117 (98.5)		49 (1.0)	
**Trial endpoints**	**Cluster mean (95% CI)**	**Cluster mean (95% CI)**	**Risk difference (95% CI) unadjusted analysis**	**p-value**	**Risk difference (95% CI) adjusted analysis** ^**2**^	**p-value**
Received an ACT:^1^ Proportion of febrile	99.8	62.5	-37.3	<0.001	-37.6	<0.001
patients receiving an ACT	(99.6 to 100.0)	(51.4 to 73.7)	(-47.5 to -27.0)	.	(-51.8 to -23.4)	.

^1^ ACT defined as receiving artemether/lumefantrine (AL) or rectal artesunate^2^ Adjusted for age, net use by patient and level of qualification of drug shop vendor

**Fig 4:**
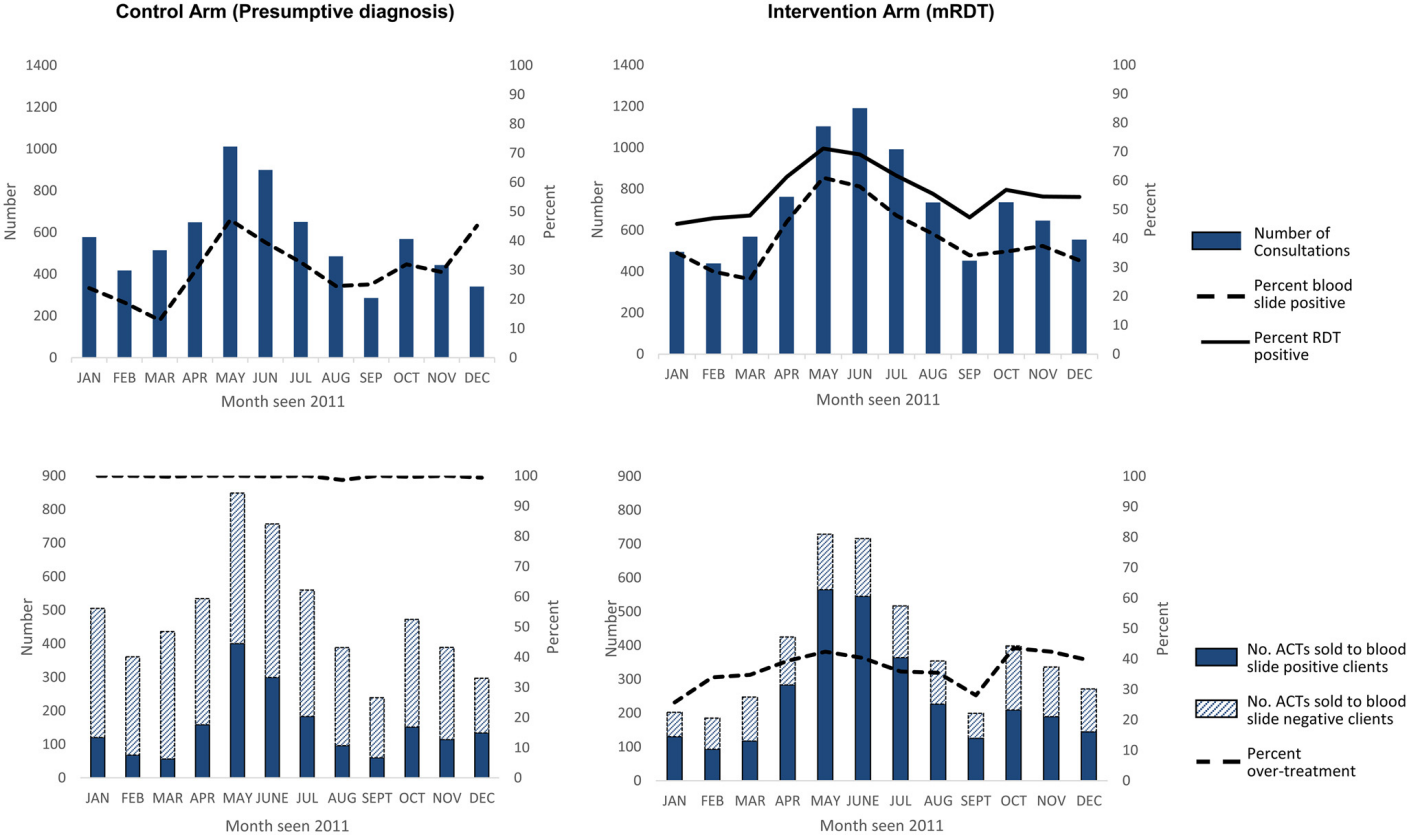
Number of consultations, malaria infection status and ACT treatment by month (Jan–Dec 2011).

**Fig 5:**
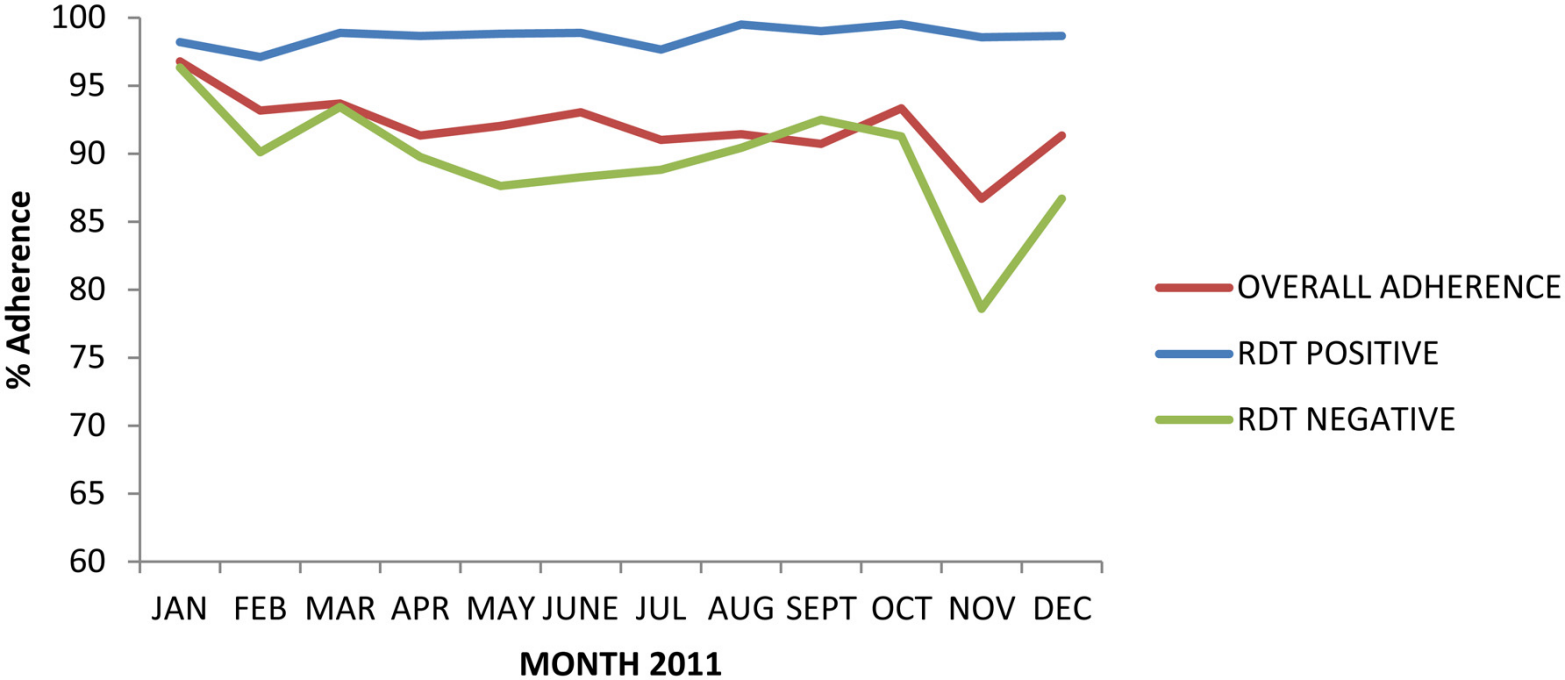
Provider adherence by month (Jan–Dec 2011).

The number of patients seen as well as the proportion of patients testing mRDT positive, peaked in May-June and October, and mirrored seasonal variation in slide positivity (Fig 4). A comparison of mRDT results to microscopy showed that mRDTs had a high sensitivity of 91.7%, but low specificity of 63.1%, with a high number of false positive mRDT results (Table 4). Overall, over a third of mRDT positive clients were parasite negative by expert microscopy, with mRDT positivity exceeding slide positivity in every month throughout the year. Adherence to mRDT results was high throughout the year and did not show any seasonal variation (Fig 5).

**Table 4:** Sensitivity and specificity of rapid diagnostic test (mRDT).

mRDT result reported by Drug Shop Vendor^1^	Expert microscopy^2^ Slide positive (%)	Expert microscopy^2^ Slide negative (%)	Predictive value of mRDT
mRDT positive	2975 (91.7)	1559 (36.9)	65.6
mRDT negative	269 (8.3)	2666 (63.1)	90.8
Total samples examined	3244	4225	

^1^ Missing data on mRDT result: 53

^2^ Results of blood slides double-read by two independent microscopists, with discrepant findings resolved by a third independent reader. All microscopy was blind to mRDT result

More patients attending drug shops on the intervention arm reported using a mosquito net the previous night, *P*.*falciparum* prevalence by light microscopy during the trial period was found to be higher in the intervention than in the control arm; 43·5% versus 31·8% (Table 5).

**Table 5:** Diagnosis and treatment of malaria at registered drug shops: Effect of mRDTs on appropriate targeting of ACTs.

	**Control arm**	**Intervention arm**				
**Diagnosis and treatment**	Frequency (%)	Frequency (%)				
Clients with complete data for trial endpoint	n = 5797	n = 7522				
Blood slide positive	1841 (31·8)	3271 (43·5)				
*Malaria treatment prescribed*						
Artemether-lumefantrine tablets (AL)	5751 (99·2)	4532 (60·2)				
Rectal artesunate suppository	36 (0·6)	47 (0·6)				
Neither AL nor rectal artesunate	10 (0·2)	2943 (39·1)				
*Malaria treatment by infection status*						
Blood slide negative, received no ACT^1^	8 (0.2)	2662 (62·6)				
Blood slide negative, received ACT	3948 (99·8)	1589 (37·4)				
Blood slide positive, received ACT	1839 (99·9)	2990 (91·4)				
Blood slide positive. received no ACT	2 (0·1)	281 (8·6)				
	**Cluster mean**	**Cluster mean**	**Risk difference (95% CI)**	**p**	**Risk difference(95% CI)**	**p**
**Trial endpoints**	**(95% CI)**	**(95% CI)**	**unadjusted analysis**	**p-value**	**adjusted analysis** ^**2**^	**p-value**
*Over-treatment with ACT*						
Proportion of blood slide negative patients receiving ACT	99·8	42·4	- 57·4%	<0·001	-72·6%	<0·001
	(99·5 to 100.0)	(28.8 to 56·0)	(-69·8 to– 44·9)		(-98·4 to– 46·7)	
*Appropriately targeted treatment*:						
Proportion of febrile patients receiving appropriate treatment of malaria with ACT	33·7	72·9	+39·3%	<0·001	+ 36·1%	<0·001
	(25·8 to 41·5)	(67·3 to 78·6)	(+30·3 to +48·2)		(+21·3 to +50·9)	
*Prompt and appropriately targeted treatment*:						
Proportion receiving appropriate treatment within 24 hours of onset of symptoms	26·8	52·8	+26·0	<0·001	+ 25·2%	<0·001
	(19·5 to 34·2)	(45·9 to 59·7)	(+16·6 to +35·2)		(+12·3 to +38·0)	

^1^ ACT defined as receiving artemether/lumefantrine (AL) or rectal artesunate

^2^ Adjusted for age, net use by patient, level of qualification of drug shop vendor, and primary endpoint (appropriately targeted treatment) at baseline.

### Effect of intervention on appropriate targeting of ACTs: mRDT-based diagnosis compared to clinical diagnosis

Examining treatment in relation to malaria infection status by microscopy, 37·4% of clients with a negative research slide in the mRDT arm had received an ACT, compared with almost 100% of blood slide negative clients over-treated with ACTs in the presumptive diagnosis arm; equating to a risk difference of -72·6% (CI -98·4–46·7), *p*<0·001, adjusted for the cluster randomised design and imbalances between arms at baseline (Table 4). Despite the high number of false positive mRDT tests, when treatment was evaluated against malaria infection status by microscopy, appropriate targeting of ACT treatment amongst patients seen in drug shops in the mRDT intervention arm was 72·9% (CI 67.3–78·6) compared with 33·7% (25·8–41·5) in the control arm; a difference of +36·1% (CI+21·3– +50.9), *p*<0·001, adjusted for cluster randomised design and imbalances between arms at baseline. The proportion of clients receiving appropriate ACT treatment within 24 hours was also significantly higher in the intervention arm, though less marked.

Malaria treatments with ACT varied by month in both arms, and were similar to seasonal variations in slide positivity (Fig 4). Exploratory analyses found that appropriate treatment varied according to age of the patient with adults less likely to receive appropriate ACT treatment than children less than five years of age (S1 and S2 Tables).

### Effect of intervention on patient outcomes and patient satisfaction

In general, few patients were referred to government health facilities, though more were referred from drug shops in the intervention arm; 11.2% (839 patients) versus 3.3% (189 patients) in the control arm, *P*<0·001.

Amongst the sub-sample of 506 patients randomly selected and followed four days after visiting a drug shop, there was no evidence to indicate that patient outcomes differed substantially between arms: 151/249 (60·6%) of patients in the intervention arm and 138/243 (56·8%) in the control arm said they had fully recovered by day 4 (self-reported). Overall, there was high patient satisfaction reported in the follow up study, with over 95% of patients in both arms saying they would visit the same DSV again. The most common response given for why they would return to the same DSV was because of a good service and/or it was the drug shop that they usually went to; while the provision of referral advice was the least commonly given response for both arms (<4%) (Table 6).

**Table 6:** Patient satisfaction (N = 506).

	Control arm	Intervention arm
	Frequency (%)	Frequency (%)
***Which aspects of the treatment and advice you received at the drug shop were you happy with*?**		
Patient recovered / treatment worked	118 (54.4)	91 (41.5)
Use of ‘blood test’ / mRDT	19 (8.8)	101 (46.1)
Standard and form of health care provided	111 (51.1)	81 (37.0)
Price of treatment and provision of credit	20 (9.2)	13 (5.9)
Facilities available and stock of medication	9 (4.1)	2 (0.9)
Offered referral and/or conditional advice	4 (1.8)	6 (2.7)
***Which aspects of the treatment and advice you received at the drug shop were you unhappy with*?**		
None	104 (84.5)	81 (71.7)
Poor standard and form of health care provided	2 (1.6)	12 (10.6)
RDT / ‘blood test’ negative result (did not agree with it)	1 (0.8)	6 (5.3)
Did not explain or show test (Blood / mRDT results)	4 (3.2)	3 (2.6)
Treatment did not work	4 (3.2)	4 (3.5)
Treatment was expensive	3 (2.4)	2 (1.8)
Did not give referral advice	0 (0)	2 (1.8)
***Was there any aspect of the treatment and advice you received that was unexpected*?**		
Collection of blood (for mRDT and/or microscopy)	21 (25)	55 (55)
Use of mRDT	5 (5.9)	37 (37)
Speed of mRDT	1 (1.2)	6 (6)
Provision of credit and low cost of treatment	18 (21.4)	11 (11)
Patient got better / treatment worked	12 (14.3)	10 (10)
Standard and form of health care provided better than expected	2 (2.4)	7 (7)
Treatment was expensive	2 (0.9)	2 (0.9)
Treatment or diagnosis (unspecified)	12 (14.3)	14 (14)
***Given your experience*, *where do you think you will go the next time your child has fever*?**		
Same DSV	199 (95.2)	206 (97.2)
Different DSV (that uses mRDT)	4 (1.9)	1 (0.5)
Different DSV (that uses presumptive diagnosis)	2 (1.0)	0 (0.0)
Public Health Facility	3 (1.4)	4 (1.9)
Other / Don’t know	1 (0.5)	1 (0.5)
***What would you recommend improving with regard to DSV*?**		
Continue mRDT use / expand to other DSVs	26 (31.7)	10 (13.3)
Develop mRDTs for other illnesses	11 (13.4)	20 (26.7)
Improve health facilities of DSVs	16 (19.5)	19 (25.3)
Greater stock and variety of medication	19 (23.2)	8 (10.7)
Cheaper medication	8 (9.8)	12 (16.0)
Show patients test results (mRDT / blood samples)	4 (4.9)	3 (4.0)
Improve geographical access	2 (2.4)	3 (4.0)
***Given your experience with the DSV*, *what reasons would you give for going back there next time*?**		
Usually go there / treatment worked	85 (41.7)	113 (54.1)
Performs a mRDT / Blood test	7 (3.4)	66 (31.6)
Provides good service and care	62 (30.4)	58 (27.7)
Geographical proximity	31 (15.2)	25 (12.0)
Low cost and/or provide credit	30 (14.7)	22 (10.5)
Stock and variety of medication available	9 (4.4)	8 (3.8)
Give referral advice	3 (1.5)	7 (3.3)

## Discussion

The results of this trial show that introducing a package of training and subsidized mRDTs in registered drug shops substantially increased appropriate treatment of malaria with ACT in the private sector; directing ACT to those patients who are parasite-positive. If mRDT were to be subsidised through mechanisms such as the Affordable Medicines Facility—malaria (AMFm) [17], improved targeting of ACT use in the private sector could reduce unnecessary waste of resources.

Adherence to the mRDT results by the drug shop vendors in the trial exceeded 85%. In any trial of provider practice there is a risk that practitioners may modify their behaviour in the knowledge that adherence to guidelines is being monitored (Hawthorne effect). To minimise this, we did not directly observe consultations or conduct exit interviews. Nor was use of simulated client visits feasible as a means of evaluating adherence to mRDT tests, since this required a blood sample to be collected from the mystery shopper. Our analysis is thus based on routine self-reported data by the drug shop vendors; however we do not consider the results observed can be attributed to reporting bias. First, there was good inter-rater agreement between used mRDTs re-read by the research team and mRDT results reported by drug shop vendors. Second, despite some evidence of possible misreporting from the small number of patient follow-up interviews conducted at the end of the first year of implementation, in which 12% of mRDT-negative clients recalled having purchased an ACT when there was no record of such in the DSVs’ records, when a computer simulation was performed to take this into account, the difference in appropriately-targeted treatment between arms decreased only slightly from +36.1% to +33.9% (SD ± 0.14), and did not substantially alter our conclusions. Third, the findings are broadly consistent with other trials, where better provider adherence and appropriately targeted treatment is seen when mRDTs are introduced into public sector settings where malaria treatment has previously been based on a presumptive clinical diagnosis [38].

We are aware that higher volumes of clients may exert different pressures on correct dispensing behaviour, especially when combined with the time to wait for the test result. However in contrast to public health facilities, the number of febrile patients consulting a registered drug shop was quite low (typically <10 per day) and did not present a major constraint. Thus, we do not consider this to have unduly affected the comparability of the two arms.

Adherence to mRDT results was however higher than previously seen in other studies in the private sector [36]. The intervention in this trial was designed to support the change in diagnostic practice amongst drug shop vendors in a number of different ways, including participatory training based on adult-learning theory, close support supervision during the initial start up period, and community sensitisation on the value of diagnostic testing. Supportive supervision has previously been shown to be effective in restricting anti-malarial drugs to parasite-positive patients amongst public sector providers [18–20]. Furthermore, a mixed methods process evaluation, carried out alongside this trial suggests that the successful uptake and adherence to diagnostic testing can largely be attributed to the fact that mRDTs were highly attractive to both patients and drug shop vendors [31, 36]. For patients, the mRDTs were a marker of accurate treatment from people with otherwise uncertain skills and credentials. Introducing mRDTs removed the ‘guesswork’ and patients believed that mRDTs enabled them to receive the correct treatment. For drug shop vendors, being trained to perform diagnostic testing using mRDTs was a stamp of approval from the Ministry of Health, and the paraphernalia of the mRDT tests aligned the DSVs more closely with the medical profession. Drug shop vendors perceived that this increased their status and they felt it had a positive impact on their business. The high acceptability of mRDT testing to patients was consistent with expressed desires by community members during the formative research at baseline for increased certainity in malaria diagnosis, and an existing awareness that not all fevers are malaria, [30, 31] which were reinforced during community sensitisation. Thus, the high desirability of mRDTs to both patients and providers may have been synergistic in creating an environment conducive to diagnostic testing. A desire to retain the increased legitimacy and newfound status that came with the introduction of blood testing may have been an additional incentive for DSVs to adhere to treatment guidelines.

Drug shop vendors in both arms benefitted from training, certification and close support supervision, and both arms received subsidized ACTs—additional factors which may also have served to increase store image, status and revenue. Whilst the availability of mRDT testing may have conferred an additional effect on store image in the intervention arm, this difference is likely to have been outweighed by the benefits of having subsidized ACTs—which were available to patients from drug shops in both arms. Thus the positive effects arising from several aspects of being in the trial may have helped incentivize drug shops to comply with treatment guidelines. Nonetheless since these benefits accrued to both intervention and control shops, we consider it unlikely that the results could be explained by a systematic difference in compliance between the two arms. There were anecdotal reports that some drug shops in control arm may have acquired and sold mRDTs, but this did not appear to be widespread. Moreover, since the effect of this type of spillover, if any, would be to dilute the intervention effect, we do not consider this to be a concern for the interpretation of our findings. Furthermore, since the two groups received exactly the same education (except for use of mRDT), we are confident that the observed difference in outcome measures can be attributed primarily to the training and use of mRDTs.

In this study we randomized clusters of drug shops in defined geographical areas. As diversity of choice and quality is characteristic of private sector provision, we did not seek to restrict patient choice. Neither can we necessarily expect that patients would go to the nearest drug shop. Registered drug shops offering mRDT testing generally saw more patients. Although there were no overt systematic differences in the characteristics of patients that chose to patronize drug shop offering mRDTs compared to those that patronized shops in the control arm; a greater proportion of patients visiting mRDT intervention shops were found to have malaria parasites (were slide-positive); a difference which could tend to overestimate the effect size than if patients in the two arms were more comparable in terms of slide positivity. Nonetheless, when the data are examined separately according to slide result, the improvement in appropriately targeted treatment appears to be due largely to reduced ACT use in slide negative patients. Furthermore, when analysis is restricted to slide negative patients, the use of mRDTs is also associated with highly marked reduction in over-use of ACTs. These results do however suggest that availability of diagnostic testing may have influenced treatment-seeking behaviour within the study area. Household surveys had been planned, but were not untaken due to financial constraints, so we are unable to fully assess if (and how) the intervention shaped consumer behaviour, nor the effect that this could have on market dynamics. Evidence from the qualitative evaluation conducted alongside this trial does however support the notion that the intervention made drug shops more attractive for care seekers, increasing clientele and sales of medication in the private retail sector, and decreasing treatment seeking from other private sector providers and public facilities. This is an area of interest we recommend for future research.

The patient population included all clients seeking treatment for fever, and therefore is representative of the population that attends drug shops. Since this was a randomised study, it is reasonable to assume that health risks of patients were similar. Since most districts in Uganda have the same rural-urban distribution of drug shops; and registration status, we think these results could be generalised to many areas in Uganda with the same endemic settings. Although this is a randomized trial and despite the strength of the evidence that such a design generates, we are fully cognizant that an intervention in registered drug shops tested under trial conditions may differ markedly from what might be observed under routine operational conditions, other types of provider, and/or other settings. Nonetheless this trial was designed and implemented by Ministry of Health with a view to realities of practice and to be reproducible at scale.

Our results are broadly in line with previous findings in Uganda; which have shown that mRDTs can be stocked and used safely to treat malaria outside formal health facilities [30, 39, 40] and that their use can lead to reduced prescription of anti-malarial drugs among mRDT negative patients. The present data shows that DSVs in the intervention arm did refer mRDT negative patients; although referral uptake was poor. A qualitative analysis of the referral process found that referral by drug vendors met with some resistance from health workers in the public sector, reflecting the uneasy liminal position that private providers occupy within the health system as a whole [36].

The sensitivity of the mRDT in this study was high at 91.7% and this compares well with previous findings in Uganda where the sensitivity for HRP2 was 92% [41]. Another study in a high malaria endemic setting showed a high sensitivity of 96.8% [42]. However, the specificity was low at 63.1% indicating potential of over-treatment with mRDTs.

Although this intervention trial was designed and implemented by Ministry of Health with a view to realities of practice and to be reproducible at scale, it was a randomized research trial. Despite the strength of the evidence that such a design generates, we recognise that an intervention in registered drug shops tested under trial conditions may differ markedly from what might be observed under routine operational conditions, other types of provider, different pricing models, profit margins, and/or other regulatory settings. Nevertheless, the results of this trial demonstrate that, when introduced as part of a comprehensive intervention, mRDTs can serve to guide better diagnosis of malaria in registered drug shops, reduce over-prescription of ACT and increase the appropriate targeting of malaria treatment with ACTs to those patients infected with malaria parasites. The results provide evidence to support the scale up mRDTs and ACTs in the private sector in Uganda and other African countries, and provide grounds for optimism that malaria treatment in the private sector can be improved.

## Supporting Information

S1 CONSORT Checklist CONSORT checklist. (DOC)

S1 Protocol Trial protocol. (DOC)

S1 Table Appropriately targeted malaria treatment in drug shops by age and sex. (DOCX)

S2 Table Prompt and appropriately targeted malaria treatment in drug shops by age and sex. (DOCX)

S1 Text Data availability. (DOCX)
