# Supplementary material for: A Cluster Randomised Trial Introducing Rapid Diagnostic Tests into Registered Drug Shops in Uganda: Impact on Appropriate Treatment of Malaria
Source: PLoS One. 2015 Jul 22;10(7):e0129545. doi: 10.1371/journal.pone.0129545 (PMC4511673; doi:10.1371/journal.pone.0129545)
Supplement: S1 Protocol — (DOC) [file pone.0129545.s002.doc]

## ACT Consortium

## Study 3: Use of RDTs in registered drug shops in Uganda

## *Revised Protocol – April 2010*

**TITLE OF PROPOSAL:**

**Strategies to improve malaria diagnosis and use of artemisinin combination therapy in the home management of malaria (HMM) in Uganda: randomized trials to evaluate the role and cost-effectiveness of rapid diagnostic tests in HMM**

**PRINCIPAL INVESTIGATOR(S) AND AFFILIATIONS:**

Anthony Mbonye, Assistant Commissioner of Health Services, Department of Community Health, Ministry of Health, Kampala, Uganda

Richard Ndyomugyenyi, Head, National Malaria Control Programme, Ministry of Health, Kampala, Uganda

Pascal Magnussen, DBL Centre for Health Research and Development, Institute of Veterinary Pathobiology, Faculty of Life Sciences, University of Copenhagen, Fredericksberg, Denmark

Siân Clarke, Department of Infectious and Tropical Diseases, London School of Hygiene and Tropical Medicine, London, UK

**PI FOR CORRESPONDENCE, WITH E-MAIL AND MOBILE PHONE:**

Siân Clarke, London School of Hygiene and Tropical Medicine, Keppel St, London,

WC1E 7HT, UK. [sian.clarke@lshtm.ac.uk](mailto:Sian.clarke@lshtm.ac.uk); tel. +44 (0)207 299 4642, + 44 (0)776 665 4045

**LEAD PI(s) WITH E-MAIL AND MOBILE PHONE, WHERE DIFFERENT FROM CORRESPONDING PI*:**

Richard Ndyomugyenyi, National Malaria Control Programme, Ministry of Health, Kampala, Uganda. [richardndyomugyenyi@yahoo.co.uk](mailto:richardndyomugyenyi@yahoo.co.uk); tel., +256 (0)772 457 980

Anthony Mbonye, Assistant Commissioner of Health Services, Department of Community Health, Ministry of Health, Kampala, Uganda. [vpadmn@infocom.co.ug](mailto:vpadm@infocom.co.ug);

tel. +256 (0)772 411 668

**COLLABORATORS AND AFFILIATIONS:**

Kristian Schultz Hansen, London School of Hygiene and Tropical Medicine, London, UK

Clare Chandler, London School of Hygiene and Tropical Medicine, London, UK

James Mugisha, Institute of Social Sciences, Makerere University, Kampala, Uganda

Harparkash Kaur, London School of Hygiene and Tropical Medicine, London, UK

**PROPOSED STARTING DATE:** March 2009

**DURATION (MONTHS):** 36 months

**TOTAL BUDGET REQUESTED:** US$ 1,659,963

**TOTAL BUDGET SPEND IN NORTHERN INSTITUTIONS* US$465,093**

**INSTITUTION WHERE GRANT TO BE HELD:**

London School of Hygiene & Tropical Medicine

**SITE(S) OF RESEARCH (COUNTRIES, LOCATIONS, INSTITUTIONS):**

- Uganda, Rukungiri District, Malaria Control Programme, Ministry of Health
- Uganda, Mukono District, Department of Community Health, Ministry of Health

**AIMS OF THE ACT CONSORTIUM THIS PROJECT ADDRESSES***

1. Improving access.

To evaluate alternative models of ACT delivery involving public sector providers, private facilities, retailers, community health workers and mothers/caretakers so as to maximise access to effective antimalarial drugs for those who have malaria.

1. Improving drug targeting.

To determine the impact on antimalarial drug use and health outcomes of interventions to improve rational drug prescribing and dispensing, including use of rapid diagnostic tests, so as to minimise over-diagnosis of malaria and over-prescription of antimalarial drugs

**SUMMARY:**

Most malaria deaths occur within 48 hours of onset of symptoms, and in rural areas with poor access to health facilities, home management of malaria (HMM) can improve the timeliness of treatment and reduce malaria mortality by up to 50%. In order to maximize both coverage and impact, artemisinin combination therapies (ACTs) should be deployed in HMM programmes, as well as in formal health facilities. Up to 80% of malaria cases are treated outside the formal health sector and shops are frequently visited as the first (and in some cases only) source of treatment. Strategies to deploy ACTs in Africa thus also need to examine the role of shops in home management and to ensure that drugs sold are appropriate. The current practice of presumptive treatment of any febrile illness as malaria (both at health facilities and in the context of HMM) based solely on clinical symptoms without routine laboratory confirmation, results in significant over-use of antimalarial drugs. With ACT being a more costly regimen, it is important to be more restrictive in its administration and rapid diagnostic tests (RDTs) provide a simple means of confirming malaria diagnosis in remote locations lacking electricity and qualified health staff.

This study therefore proposes to evaluate the feasibility, acceptability, and cost-effectiveness of using RDTs to improve malaria diagnosis and treatment by two types of community-based providers:

(a) community drug distributors and (b) local drug shops.

The accuracy of RDTs, and the acceptability of this approach, will be evaluated in both low and high transmission areas.

**RESEARCH PROPOSAL (Maximum 10 pages - font size 11):**

**(a) Background to the study. Maximum 1 page**

The main component of the current malaria control strategy in Sub-Saharan Africa is to reduce malaria-related mortality and severe morbidity through early diagnosis and effective case management. The majority of *Plasmodium falciparum* malaria deaths occur within 48 hours of onset of symptoms, and in rural areas with poor access to health facilities treatment is often given too late. Home management of malaria (HMM) improves the timeliness of treatment and has been shown in field trials to reduce malaria mortality by up to 50%. Large-scale research studies have also shown that scaling up HMM is both feasible and effective.^1^ Strategies to deploy ACTs need to investigate the feasibility of incorporating the use of ACTs into HMM programmes, in order to maximize both coverage and impact on malaria morbidity and mortality.

In much of Africa, up to 80% of malaria cases are treated outside the formal health sector and polypharmacy is common.^2^ Factors affecting low utilization of formal health facilities include distance, waiting time, disrespectful behaviour of staff towards patients, and drug stockouts. In comparison, shops selling antimalarial drugs are more numerous, more easily accessible, and more oriented to satisfying consumer needs. As such, these shops are frequently visited as the first (and in some cases the only) source of treatment.^3-5^ Strategies to deploy ACTs in Africa also need to take into account the role of shops in home management of malaria and to ensure that drugs sold are appropriate.

The current practice of presumptive treatment of any febrile illness as malaria (both at health facilities and in the context of HMM) is based solely on clinical symptoms without routine laboratory confirmation and results in over-use of antimalarial drugs.^6^ The higher cost of ACTs and uncertainties that supply can meet global demand necessitates a change in diagnostic approach to reduce over-diagnosis and misuse of the new generation of antimalarial drugs.^7-10^ Rapid diagnostic tests (RDTs) can be used to confirm malaria infection and are very simple to use, require minimal training and may prove cost-effective in the context of ACT.^11-13^ With the majority of malaria cases being ‘self-diagnosed’ by the patient and treated with drugs purchased from a local shop,^3,4^ changes in diagnostic practice are not only needed amongst health-care providers, but also amongst patients themselves, pharmacies and other drug sellers. The availability of diagnostic confirmation in drug shops may increase willingness to pay for new ACT treatments. Reducing over-prescription of costly ACTs should offset the increased costs of diagnosis, however the cost-effectiveness of this approach will depend largely on the local prevalence of malaria.

We therefore propose to examine the cost-effectiveness of ACT in the home management of malaria (HMM-ACT) in a range of transmission settings, including both high and low transmission, comparing two approaches:

1. current practice where HMM is based on clinical symptoms without any laboratory confirmation, and
2. HMM following rapid diagnostic test (RDT).

The research will be undertaken in two geographically distinct but complementary studies, which will investigate the cost-effectiveness of introducing RDTs into:

- HMM-ACT administered by trained community-based distributors, in two districts of rural Uganda to compare cost-effectiveness in an area of high, perennial transmission and an epidemic-prone area of low and seasonal transmission.
- Local drug shops in a rural district of Uganda with a wide choice of treatment providers.

This proposal thus comprises of two work packages. The second work package in which the use of RDTs by shop keepers is evaluated is described below.

In both studies clinical, economic and socio-behavioural analyses will be undertaken to provide a comprehensive evaluation of the interventions to improve quality of diagnosis and treatment. Methods of evaluation will be standardized across the two sub-studies to ensure comparability of findings. The results of these studies will help policy makers to make a decision on the deployment of ACTs and RDTs in home-based management of malaria. The data generated will help identify the most appropriate strategy for a range for epidemiological settings. Research findings will also help identify how to achieve maximal coverage of correct and appropriate malarial treatment and to minimize misuse of this valuable new drug combination.

WORK PACKAGE II:

Introducing rapid diagnostic tests (RDTs) into the private health sector in Uganda: a randomised trial among registered drug shops to evaluate impact on antimalarial drug use

**Lead PI:** Dr Anthony Mbonye

**(b) Aim and objectives**

**Aim:** This study aims to examine the feasibility of introducing RDTs into registered drug shops in Uganda to encourage rational drug use in case management of malaria, with a focus on the correct use and adherence to ACTs. Study outcomes will assess drug prescription practices in drug shops and public health services, as well as treatment-seeking behaviour by local communities, including sources of drug treatment, consumer preferences, demand for improved diagnosis, and use of ACTs. The study also examines differences in utilization between households and ability of the approach to improve quality of diagnosis and treatment, especially amongst the poorest members of society.

It is hypothesised that the use of diagnostic testing will reduce over-diagnosis and over-prescription of antimalarial drugs in the private sector. It is further hypothesised that the availability of diagnostic testing in drug shops and confirmation of malaria will increase uptake and sale of ACT combinations (rather than monotherapy or cheaper, less efficacious, antimalarial drug combinations), and may also encourage purchase of the full treatment dose, as well as patient adherence to the full course of ACT treatment.

**Primary objectives**

1. To evaluate the impact of availability of RDTs in the commercial sector

- on appropriate antimalarial drug sales in the commercial sector (incl. consumer demand, treatment advice given in registered drug shops)
- on appropriate antimalarial drug prescription in cases referred to the formal health sector

1. To describe and quantify treatment-seeking behaviour under a scenario of ACT provision in both the commercial and formal health sectors, including identification of choice menus, initial source of treatment, antimalarial drugs used and economic costs.

**Secondary objectives**

1. To assess perceptions and acceptability of the use of RDTs among malaria patients and drug providers.
2. To assess the impact of improved malaria diagnosis on ACT adherence among providers and consumers.

**(c) Study design and methods**

***Study design***

A cluster-randomized trial of RDTs in registered drug shops in a rural area of Mukono District, an area of high transmission in Central Uganda. Numerous sources of malaria treatment can be found in the area, including government health facilities, licensed pharmacies, trained community distributors of pre-packaged drugs (Homapak) for home management of malaria, registered drug shops and unlicensed shops selling drugs.

### As the study aims to evaluate consumer preferences, treatment actions and demand for improved malaria diagnosis in the private sector, treatment options are designed to mimic current choices. Communities resident within the study area will thus be able to seek ACT treatment from a range of providers including government health facilities, local pharmacies and registered drug shops (some of which will offer improved diagnosis through use of a RDT), as shown in the table below.

### The study will be undertaken in two sub-counties, and census enumeration areas will be randomized to either intervention or control. Registered drug shops located in intervention areas will receive training in the use of rapid diagnostic test for malaria (RDTs) and supplied with RDTs by the research team, and trained to only recommend antimalarial treatment with Coartem® after a positive test result. In the control areas, registered drug shops will continue to dispense antimalarial treatment based on presumptive diagnosis (current practice).

### RDTs have recently been introduced into government health facilities in Uganda. Government health staff in the study area will therefore receive additional supportive training to use RDTs and to only provide treatment with Coartem® after a positive result. The study will ensure that public health facilities are well stocked with RDTs, antimalarials and other essential drugs through the government credit supply system. Community sensitization meetings will be carried out to increase awareness of ACTs, reasons for the new treatment policy for malaria, the need for better diagnosis, and importance of adherence to treatment.

| **Private facilities (pharmacies and drug shops)** |
| --- |
| Drug shops randomised to either:  1) Confirmatory diagnosis with RDTs, followed by sale of a commercially-available ACTs only to those who have a positive RDTs  **or**  2) Current practice:  Presumptive clinical diagnosis and treatment of fever |

*Key strengths to the study site*

- - Multiple sampling points within the same district and health system
  - Study design allows comparison between private and public health care providers to assess patient preferences

This allows direct comparison of the effectiveness of treatment, and the cost-effectiveness of improved diagnosis, in a range of different health care providers within the same country.

*Primary endpoints*

- Proportion of patients receiving appropriate treatment from a commercial drug outlet: % of patients diagnosed with malaria who receive appropriate[[1]](#footnote-2) ACT treatment (irrespective of time since onset of treatment).
- Over-prescription: % of patients, who are not parasite-positive, who receive inappropriate ACT treatment from a commercial drug outlet.
- Provider adherence to RDT result: % of patients who receive appropriate treatment, consistent with RDT result
- Cost-effectiveness of RDT compared to presumptive treatment in home management of uncomplicated malaria.

All endpoints will be examined and reported according to age and sex: children aged 0-4y, 5-15y and adults ≥ 16y. For each age group, the proportion of patients receiving appropriate treatment will be compared between shops with RDTs and shops without RDTs, in order to determine whether diagnostic testing reduces overuse of antimalarials in that age group.

### *Secondary endpoints*

- Proportion of patients receiving prompt effective treatment from a commercial drug outlet: % of patients diagnosed with malaria who receive appropriate^2^ ACT treatment within 24 hours of onset of malaria.
- Acceptability of RDTs: % of patients offered a diagnostic test, % of ACT sales preceded by a test
- Perceptions of ACTs and other antimalarial drugs (amongst community; drug sellers; health staff)
- Perceptions and acceptability of malaria diagnosis using RDTs (amongst community; drug sellers; health staff)
- Willingness to offer and perform RDTs to malaria patients (among drug sellers, health workers)
- Patient adherence to prescription (complete dose taken by day 4)
- Referral: Appropriate treatment of RDT negative patients (lack of antimalarial sale and provision of referral); Timeliness and uptake of referral at a health unit by referred patients.
- Coverage of prompt effective treatment: % of patients with fever who received ACT treatment within 24 hours of onset of malaria, measured through household surveys. For comparison with the WHO/RBM indicator in national and international statistics, coverage in children <5 years will be reported separately from older children and adults.
- Source of treatment: % of patients attending health facility for treatment; % visiting a drug shop
- Equity of diagnosis: % of malaria patients receiving a diagnostic test for malaria, across socio-economic groups.
- Equity of treatment: % of patients, who are diagnosed with malaria, receiving treatment with an ACT, across socio-economic groups.

***Methods***

*Diagnosis and treatment procedures*

Private providers will be trained on how to recognize and distinguish uncomplicated & severe malaria, supplying unit-dose packaged Coartem® to customers with uncomplicated malaria, and administration of rectal artesunate pre-referral treatment and referral for children with severe and complicated malaria. Rectal artesunate will be provided free of charge for children with severe malaria who cannot take oral medication. Shops will be provided with low literacy visual aids to help explain the symptoms, diagnosis, correct treatment of malaria and danger signs. The shopkeepers will explain the pictorial instructions to customers, as well as give instructions on dosing and how to give the drug to the patient.

Private providers in the intervention areas will be trained in use and interpretation of the rapid diagnostic test. Shops will be supplied with RDTs and a placard identifying them as an approved provider of malaria diagnostic tests. Private providers will be allowed to sell the test, at a price determined by pre-intervention willingness-to-pay studies. The age and sex of patient, onset of symptoms and promptness of treatment-seeking, diagnosis (including RDT result, where appropriate), tablets supplied and outcome of treatment will be recorded by dispensers. Drug sellers will also be trained in the preparation of thick blood films for malaria, and a research blood slide will be prepared from all patients being treated with an antimalarial drug (irrespective of use of RDT) for later validation of diagnosis and treatment by microscopy. All drug sellers will be trained on good laboratory practices, proper disposal of sharps etc. Sharps containers will be provided, and collected regularly for correct disposal at the health facility by the research team.

Consent will be obtained from drug shop owners to participate in the trial, and for randomization of shops to receive RDTs.

Patients are free to choose which shops they use, and will be able to choose between outlets that offer diagnostic testing and those that do not, as this service will be openly advertised. Patients seeking treatment for uncomplicated malaria (or fever) from a registered drug shop in the intervention areas will be offered a RDT prior to drug purchase. Verbal consent will be sought from patients accepting the test, to also prepare a blood film from the fingerprick blood sample for the purposes of the research. Patients who decline an RDT test (and patients seeking treatment at registered drug shops in non-intervention areas) will receive presumptive treatment, as per current practice. This outcome (no test) will be recorded and whether an antimalarial was purchased or not.

Community sensitization on the need for treatment of febrile illness within 24 hrs after onset of illness will also be carried out. Sensitization meetings will also aim to increase awareness of ACTs within the community, the need for better diagnosis, and the importance of adherence to treatment.

*Referral mechanism of severe malaria and non-malarial fevers*

Severe malaria: Children with danger signs consistent with suspected severe malaria (WHO definition 2000) will be given pre-referral treatment using a rectal artesunate suppository and referred immediately to the nearest health unit for further treatment. Patients will be referred using an emergency referral form, including details of symptoms and the use of pre-referral treatment. Shops will keep a dated register for referrals and copy of the referral form with personal identifiers (also at health units).

Non-malarial fevers: By law, registered class C drug shops are not permitted to stock antibiotics or other “prescription only” medicines. Commercial drug sellers will therefore also be trained to examine and refer febrile patients with fever (axillary temperature >37.0^O^C and a negative RDT (i.e. non-malaria) to the nearest health facility for examination and treatment by a fully qualified health worker, where appropriate. Any patient with danger signs and/or a temperature >38.5^O^C and a negative RDT should be referred to the nearest health unit as an urgent case. This additional temperature criterion is based on the WHO definition of hyperthermia and is intended to help ensure that those with severe bacterial infections are seen at a health unit. These patients will also be referred using the emergency referral form, which includes details of symptoms and the use of prior treatment. Shops will keep a dated register for referrals and copy of the referral form with personal identifiers (also at health units).

Shopkeepers will be trained to advise RDT negative patients with fever (axillary temperature ≥ 37.0^O^C) or other symptoms to seek treatment at the nearest health facility for further evaluation by a fully qualified health worker. A standard referral form will be provided to the patient for this purpose. Where no other symptoms are present, the patient will be advised on tepid sponging, but to return for further assessment at the drug shop or a health facility should the symptoms persist or worsen.

To minimise the risk of inadvertent exposure to ACTs during pregnancy, drug sellers will be trained to administer and RDT test, and to refer RDT-positive pregnant women (or women that suspect they may be pregnant) to a health facility for examination by a midwife prior to antimalarial treatment.

***Evaluation***

*Appropriateness of treatment and/or referral*

Prescription patterns and drug sales in private shops and clinics will be measured through the use of exit interviews, drug sales records and mystery shopper visits. Drug sales records will be adapted to include data on RDT results. Semi-structured exit interviews will be used to ascertain whether diagnostic testing was offered, was accepted by the patient, and type and quantity of drugs sold. Exit interviews will also investigate the patient’s perception of diagnostic testing and their knowledge and understanding of the test result, treatment advice and/or referral. In non-intervention areas, a finger-prick blood sample will be taken to prepare a research blood film to ascertain by microscopy whether the fever was due to malaria. A random sample of patients will also be followed at home after several days to ascertain adherence to treatment (see section below), outcome of the illness and total household expenditure on treatment. Patients will be recruited on exit from the drug outlet and individual informed consent will be sought from patients (parents or guardians, in the case of minors) to participate in an exit interview and any subsequent household visits.

The appropriateness of treatment provided will be evaluated as the proportion of correctly treated malaria cases, based on the “gold standard” of the research blood slide read by an experienced research technician. Appropriate treatment of fever is a composite definition including both a) those who a slide-positive who are given a 1^st^-line antimalarial and b) those who are slide-negative who are not given a 1^st^-line antimalarial. Over-prescription will also be assessed as the proportion of children who are not parasite positive who receive inappropriate ACT treatment.

The perceptions and acceptability of the use of RDT prior to malaria treatment will also be evaluated among both users and providers of treatment services through a series of focus group discussions held with commercial drug sellers and local community members. The cost of diagnostic testing in commercial outlets may well influence acceptability and will be an important parameter to investigate. The perceived trade-off between the risk of paying for diagnosis compared to the cost of paying for inappropriate and thus ineffective medicines in the eyes of the local community will be critical to the acceptability of diagnostic testing, and may also change in light of actual experience over time. Willingness to pay and other factors affecting acceptability will be investigated through exit interviews, in-depth interviews with household heads and focus group discussions. Interviews and group discussions will be conducted before and after the introduction of diagnostic testing. Informed consent will be sought from all informants prior to undertaking any interviews (focus group discussions, exit interviews, household surveys, etc.).

Household surveys will be undertaken to collect information on treatment–seeking behaviour and associated costs, primary access point for ACTs, and consumer preferences. Data on the socioeconomic profile of households will also be obtained. The geolocation of households and local treatment providers will be mapped using handheld global positioning system (GPS). The study will be conducted over a period of 24 months.

*Adherence with treatment*

Knowledge of patient adherence to the course of medication is critical in evaluating the effectiveness and appropriate deployment of ACTs. Incomplete adherence may help accelerate the development of drug resistance. Poor adherence to medication has been previously described for chloroquine and other drugs, and is likely to occur also for ACTs. Caretakers may forget to give the dose on some days or may stop medication if they perceive that patient is cured after one or two doses. In particular however, the higher cost of ACTs may cause families stop medication after one or two doses to save tablets for later use, rather than complete the full course of treatment, or patients may only purchase a few tablets (partial dose) in order to save money.

Adherence to medication will be examined in a random sample of drug store clients recruited on exit from the drug outlet. Patients will be followed-up in their homes on day 4 to check on adherence to medication. The blister pack will be examined for any remaining tablets to assess compliance, using the following definition:

- *Total:* verbal confirmation of completion of all doses in presence of an empty blister pack and a correct description of how the dose was taken.
- *Probable:* confirmation of completion of all doses in the absence of a blister pack and a correct description of how the dose was taken.
- *Non-compliance*: presence of tablets on the blister pack on Day 4 and or inability to explain how the doses were supposed to be taken

In addition, in a randomly-selected sub-sample of 600 patients, a 100µl finger-prick blood sample on day 4 will be pipetted onto filter paper to measure drug levels by HPLC method to confirm adherence. As the presence of artemether derivatives in the bloodstream is shortlived and thus difficult to measure, adherence will be based on levels of lumefantrine alone. Furthermore as lumefantrine is taken on every day of treatment (whereas artemether is only given on day one) this will provide a measure of complete adherence up to and including day three.

*Measuring the economic impact of intervention*

Cost and cost-effectiveness: To determine relative cost-effectiveness requires information on resource use (costs) as well as effectiveness of each intervention arm. Analyses will be undertaken form a societal perspective and costs will include a) those related to deployment of the intervention and borne by the health service, and b) those incurred to the household when seeking treatment for fever, including the direct costs of transport, diagnosis and drugs, and opportunity costs of lost time and production. Both recurrent and capital costs to the provider of training staff and delivering the intervention will be estimated in both scenarios of private and public providers. Resource savings to providers and households will also be assessed. Household costs will be captured through a standardised questionnaire administered during periodic household surveys to capture data on treatments sought for febrile children in the 14 days prior to the survey, and will include a minimum of 500 questionnaires. Household surveys will be carried out during the malaria transmission season, and will be repeated at least twice, pre- and post-intervention. If resources allow, more frequent surveys will be carried out to examine seasonal variations in expenditure. The unit costs to the provider of malaria treatment will be estimated at a sample of health facilities and CDDs using a combination of standard step-down costing methodology and micro-costing.

Data on the incremental cost-effectiveness of adding RDT to presumptive treatment will be generated through a pairwise comparison of groups receiving ACT treatment after a positive RDT with presumptive treatment only. Net cost-effectiveness will be calculated by deducting resources saved from the total intervention cost per case of inappropriate treatment averted.

Cost-effectiveness will vary according to levels of community uptake and compliance under different implementation conditions. Furthermore, many of the parameters included in cost-effectiveness calculations are uncertain and variable (e.g. probability of death, unit costs of drugs, unit costs of diagnostic tests, and other inputs). Sensitivity analysis will therefore be conducted on the values of key parameters, in order to explore varying scenarios and to generate a range of potential cost-effectiveness ratios.

Coverage & Equity: Coverage of prompt effective treatment within 24 hours of onset of fever will be measured through household surveys in a random sample of communities, using a 14-day recall of fever cases in the household to obtain data on treatment-seeking behaviour. The equity of access to enhanced diagnosis in the treatment of malaria in young children will be assessed in terms of total levels of utilization, as well as proportional utilization of the public and private sector, across socio-economic groups. Socio-economic status will be examined in terms of wealth, based on ownership of household and agricultural assets. Utilization will also be compared between households differing in their educational background; specific knowledge of malaria and its treatment; and distance from nearest treatment provider. The equity of provision of treatment with/without improved diagnosis will be also compared according to levels of utilization of public provider (government health units) and private providers (pharmacies and drug shops).

### *Sample size requirements*

### Sample size requirements for a cluster-randomised trial^15^ were calculated for the primary endpoint of the proportion of patients receiving appropriate treatment (in line with parasitological status). Appropriate treatment is defined as a composite including both a) those who are slide-positive who purchase an ACT and b) those who are slide-negative who do not purchase an ACT or other antimalarial drug. As the priority for improving treatment in the private sector is to increase the proportion of true cases that purchase and use an ACT (as opposed to antipyretics only, without any antimalarial treatment), we do not include prompt appropriate treatment within 24 hours as the primary endpoint in this work package.

### Studies indicate that less than 40% of adults and less than 25% of children visiting a drug shop will purchase an antimalarial.^3,4^ Conservatively assuming a baseline level of ACT purchase in 50% of consultations, sample size was calculated to demonstrate an increase in appropriate purchase of an ACT among RDT-positive patients to 75%, and a decrease in inappropriate treatment among RDT-negative patients to 20%. A cluster-randomised trial with 90% power at the 95% significance level and variation between clusters k=0.25, will require a total of 10 clusters per arm, with 61 consultations per cluster (a total of 610 consultations in each arm). A minimum of 620 patients within each age group will thus be recruited at participating outlets in each arm, yielding an overall total of 1240 patients aged 0-4y; 1240 aged 5-14y; and 1240 adults (a total of approximately 375 patients per outlet). Sample size calculations, repeated for lower levels of antimalarial purchase at baseline, required <10 clusters per arm.

The unit of randomization is the community, defined as the census enumeration area. The study will thus be conducted in 20 enumeration areas in two neighbouring sub-counties within Mukono District. Registered drug outlets located in the 10 enumeration areas randomized to the intervention will be trained in the use of RDTs, and drug outlets located in the other 10 enumeration areas will continue their current practice (presumptive diagnosis and treatment).

**(d) Essential references**

1. WHO. (2004). Scaling up home management of malaria: from research to implementation. Geneva: WHO
2. McCombie SC. (2002). Self treatment for malaria: the evidence and methodological issues. *Health Policy and Planning,* **17**: 333-344.
3. Kachur P *et al.* (2006). Prevalence of malaria parasitaemia among clients seeking treatment for fever or malaria at drug stores in rural Tanzania 2004. *Tropical Medicine & International Health*, **11 (4)**: 441-451.
4. Abuya TO *et al.* (2007). Use of over-the-counter malaria medicines in children and adults in three districts in Kenya: implications for private medicine retail interventions. *Malaria Journal* **6**: 57.
5. Kindermans J-M *et al.* (2007). Estimating antimalarial drug consumption in Africa before the switch to artemisinin-based combination therapies (ACTs). *Malaria Journal* **6**: 91
6. Reyburn H *et al.* (2004). Over-diagnosis of malaria patients with severe febrile illness in Tanzania: a prospective study. *British Medical Journal*, **329**: 1212-1218
7. Barnish G, Bates I & Iboro J. (2004). Newer drug combinations for malaria may be impractical unless diagnostic accuracy can be improved. *British Medical Journal*, **328**: 1511-1512
8. Amexo M, Tolhurst R, Barnish G & Bates I. (2004) Malaria misdiagnosis: effects on the poor and vulnerable. *Lancet*, **364 (9448)**: 1896-1898
9. D’Alessandro U, Talisuna A & Boelaert M. (2005). Should artemisinin-based combination treatment be used in the home-based management of malaria? *Tropical Medicine & International Health*, **10 (1)**: 1-2.
10. Reyburn H *et al*. (2007). Rapid diagnostic tests compared with malaria microscopy for guiding outpatient febrile illness in Tanzania: randomised trial. *British Medical Journal*, **334**: 403-409
11. WHO. (2006). The use of malaria rapid diagnostic tests. 2^nd^ ed. Geneva: WHO (WHO-TDR/WHO-WPRO 2006.)
12. Shillcutt S *et al*. (2008). Cost-effectiveness of malaria diagnostic methods in sub-Saharan Africa in an era of combination therapy. *Bulletin of the World Health Organisation*, **86 (2)**: 101-110.
13. Lubell *et al.* (2008). An interactive model for the assessment of the economic costs and benefits of different rapid diagnostic tests for malaria. *Malaria Journal* **7**: 21
14. Kallander *et al.* (2008). Delayed care seeking for fatal pneumonia in children aged under five years in Uganda: a case-series study. *Bulletin of the World Health Organisation*, **86 (5)**: 332-338
15. Hayes RJ & Bennett S. (1999). Simple sample size calculation for cluster-randomized trials. *International Journal of Epidemiology* **28**: 319-326.
16. Nsungwa-Sabiiti J *et al.* (2005). Community effectiveness of malaria treatment in Uganda – a long way to Abuja targets. *Annals of Tropical Paediatrics*, **25**: 91-100.

**CONTRIBUTION TO THE OBJECTIVES OF THE ACT CONSORTIUM**

**Indicate how this proposal meets the overall objectives of the Consortium under each of the objectives from the front page above (maximum 100 words per objective)**

1. **Improving access.**

This project examines the role of community-based treatment providers in improving access to ACTs, and the feasibility and cost-effectiveness of interventions at the community-level to improve malaria diagnosis, appropriate treatment and referral. The research proposed examines the role of two different types of providers in maximising access to prompt effective treatment at the community level: village-based community drug distributors (an extension of the public health sector) and commercial drug shops and pharmacies (private sector). The project evaluates the impact of introducing confirmatory diagnosis at the community level in increasing the proportion of cases that receive ACT treatment, and in reduced delay in seeking treatment.

1. **Improving drug targeting.**

This project examines the feasibility and cost-effectiveness of using rapid diagnostic tests (RDTs) to improve ACT targeting at the community level. The project will investigate the impact of RDT use on rational drug prescribing and dispensing of ACTs by two key providers of malaria treatment: 1) village-based community drug distributors and 2) commercial drug shops and pharmacies. In addition to impact of diagnostic testing on over-diagnosis and over-prescription; the study will also examine whether diagnostic testing in the private sector results in improved drug targeting through most frequent purchase of the correct drug, in the correct quantity, and patient adherence to the full course of treatment.

**ETHICAL ISSUES**

**Discuss ethical aspects of the proposal.**

*Treatment & referral:*

Patients seeking treatment for fever from a registered drug shop in the intervention areas will be offered a RDT prior to drug purchase. Patients declining a test (and patients seeking treatment at registered drug shops in non-intervention areas) will receive presumptive treatment, in accordance with national guidelines for home-management of malaria (current practice).

Shopkeepers will be trained in taking a fingerprick blood sample, and sterile precautions. Sharps containers will be provided, and collected regularly for correct disposal at the health facility by the research team.

Shopkeepers will be trained on how to recognize and distinguish uncomplicated & severe malaria, supplying unit-dose packaged Coartem® for patients with uncomplicated malaria and the administration of rectal artesunate pre-referral treatment to children with severe and complicated malaria. Cases of severe malaria will be referred immediately to the nearest health unit. In case of a febrile patient with a negative RDT (i.e. non-malaria), those with danger signs and/or axillary temperature >38.5 will also be referred to the nearest health unit. This additional temperature criterion is based on the WHO definition of hyperthermia and is intended to help ensure that patients with severe bacterial infections are seen at a health unit.

To minimise the risk of inadvertent exposure to ACTs during pregnancy, drug sellers will be trained to administer and RDT test, and to refer RDT-positive pregnant women (or women that suspect they may be pregnant) to a health facility for examination by a midwife prior to antimalarial treatment.

*Informed consent:*

Consent will be obtained from drug shop owners to participate in the trial, for randomization of shops to receive RDTs, to keep records on diagnosis and drug sales, and to make these available to the research team. Meetings will be held with the communities in the study areas to describe the purpose of the study, the procedures to be followed, and the risks and benefits of participation.

Patients are free to choose which source of treatment they use, including government health facilities which perform RDT testing, registered drug shops that offer RDT testing and others which use presumptive treatment. Furthermore, it is the patient’s choice which shops they use, and they will be able to choose between outlets that offer diagnostic testing and those that do not, as this service will be openly advertised. Patients seeking treatment for fever from a registered drug shop in the intervention areas will be offered a RDT prior to drug purchase, and have the right to decline the test.

Patients will be recruited on exit from the drug outlet. Individual informed consent will be sought from patients (parents or guardians, in the case of minors) to participate in an exit interview and subsequent follow-up visits at home in order to evaluate the impact of the intervention on malaria treatment. Patients will be informed that participation in the study is completely voluntary and that they may withdraw from the study at any time. Informed consent will also be sought from all informants prior to undertaking any other interviews (focus group discussions, household surveys, etc.).

**Submission to ethics committee(s) (specify which committees it will be submitted to)**

Uganda Council for Medicine, Science and Technology, Uganda

Ethics Committee, London School of Hygiene & Tropical Medicine, UK

**OPEN ACCESS AND PUBLIC HEALTH LIAISON GROUP (PHLG).**

**Submitting this proposal means investigators accept**

**a) the commitment to open access to data produced in research funded by the ACT Consortium agreed at the PIs meeting (available on the ACT Consortium Website once ratified by all PIs)**

**b) that as a condition of accepting funds through the ACT Consortium data partly or wholly collected from research funded by the ACT Consortium will be made available to the Public Health Liaison Group within a timescale agreed prospectively by the PI of the project, the Chair of the PHLG and the Secretariat, which will be part of the contract between the ACT Consortium and the institution where the grant will be held.**

**To signify agreement to this**

**Type initials of corresponding PI with date:** SC 09/05/2008

**Type initials of lead PI(s)* with date:** RN 09/05/2008; AM 09/05/2008

1. The proportion of correctly treated malaria cases is based on the “gold standard” of a blood slide read by an experienced research technician. Appropriate treatment is a composite definition including both a) those who a slide-positive who are given a 1^st^-line antimalarial and b) those who are slide-negative who are not given a 1^st^-line antimalarial (irrespective of dosing). [↑](#footnote-ref-2)
