# Supplementary material for: A Cluster Randomised Trial Introducing Rapid Diagnostic Tests into Registered Drug Shops in Uganda: Impact on Appropriate Treatment of Malaria
Source: PLoS One. 2015 Jul 22;10(7):e0129545. doi: 10.1371/journal.pone.0129545 (PMC4511673; doi:10.1371/journal.pone.0129545)
Supplement: S1 Table — (DOCX) [file pone.0129545.s003.docx]

|  | | | | | | | | |
| --- | --- | --- | --- | --- | --- | --- | --- | --- |
| Table S1. Appropriately targeted malaria treatment in drug shops by age and sex | | | | | | | | |
| Drugs shops using mRDTs | | | |  |  |  |  |  |
|  | Age group | n | Appropriate treatment | % | Odds ratio | 95% CI | | p |
|  | 0-5y | 2860 | 2221 | 77.7 | 1 |  |  |  |
|  | 5-16y | 1959 | 1393 | 71.1 | 0.72 | 0.63 | 0.83 | <0.001 |
|  | 16-60y | 2475 | 1847 | 74.6 | 0.85 | 0.75 | 0.97 |  |
|  | 60y+ | 163 | 130 | 79.8 | 1.11 | 0.75 | 1.65 |  |
|  | Male | 3632 | 2692 | 74.1 | 1 |  |  | 0.043 |
|  | Female | 3878 | 2953 | 76.1 | 1.12 | 1.00 | 1.24 |  |
|  |  |  |  |  |  |  |  |  |
| Drug shops using clinical diagnosis | | | |  |  |  |  |  |
|  | Age group | n | Appropriate treatment | % | Odds ratio | 95% CI | | p |
|  | 0-5y | 2062 | 708 | 34.3 | 1 |  |  |  |
|  | 5-16y | 1900 | 726 | 38.2 | 1.21 | 1.06 | 1.38 | <0.001 |
|  | 16-60y | 1682 | 384 | 22.8 | 0.59 | 0.51 | 0.69 |  |
|  | 60y+ | 100 | 21 | 21.0 | 0.45 | 0.28 | 0.74 |  |
|  | Male | 3947 | 898 | 22.8 | 1 |  |  | 0.785 |
|  | Female | 1846 | 948 | 51.4 | 0.98 | 0.88 | 1.10 |  |
|  |  |  |  |  |  |  |  |  |

|  |  |  |  |  |  |  |  |  |
| --- | --- | --- | --- | --- | --- | --- | --- | --- |
