# Supplementary material for: A Cluster Randomised Trial Introducing Rapid Diagnostic Tests into Registered Drug Shops in Uganda: Impact on Appropriate Treatment of Malaria
Source: PLoS One. 2015 Jul 22;10(7):e0129545. doi: 10.1371/journal.pone.0129545 (PMC4511673; doi:10.1371/journal.pone.0129545)
Supplement: S2 Table — (DOCX) [file pone.0129545.s004.docx]

|  |  |  |  |  |  |  |  |  |
| --- | --- | --- | --- | --- | --- | --- | --- | --- |
| Table S2. Prompt and appropriately targeted malaria treatment in drug shops by age and sex | | | | | | | | |
| Drugs shops using mRDTs | | | |  |  |  |  |  |
|  | Age group | n | Prompt appropriate treatment | % | Odds ratio | 95% CI | | p |
|  | 0-5y | 2837 | 1527 | 53.8 | 1 |  |  |  |
|  | 5-16y | 1945 | 1013 | 52.1 | 0.84 | 0.74 | 0.94 | <0.001 |
|  | 16-60y | 2458 | 1209 | 49.2 | 0.76 | 0.68 | 0.85 |  |
|  | 60y+ | 163 | 60 | 36.8 | 0.51 | 0.37 | 0.71 |  |
|  | Male | 3605 | 1887 | 52.3 | 1 |  |  | 0.337 |
|  | Female | 3851 | 1961 | 50.9 | 0.96 | 0.87 | 1.05 |  |
|  |  |  |  |  |  |  |  |  |
| Drug shops using clinical diagnosis | | | |  |  |  |  |  |
|  | Age group | n | Prompt appropriate treatment | % | Odds ratio | 95% CI | | p |
|  | 0-5y | 2055 | 606 | 29.5 | 1 |  |  |  |
|  | 5-16y | 1892 | 584 | 30.9 | 1.08 | 0.94 | 1.24 | <0.001 |
|  | 16-60y | 1678 | 313 | 18.7 | 0.56 | 0.48 | 0.65 |  |
|  | 60y+ | 100 | 16 | 16.0 | 0.40 | 0.23 | 0.70 |  |
|  | Male | 3605 | 1887 | 52.3 | 1 |  |  | 0.605 |
|  | Female | 3851 | 1961 | 50.9 | 1.03 | 0.92 | 1.16 |  |
