# Supplementary material for: A Cluster Randomised Trial Introducing Rapid Diagnostic Tests into Registered Drug Shops in Uganda: Impact on Appropriate Treatment of Malaria
Source: PLoS One. 2015 Jul 22;10(7):e0129545. doi: 10.1371/journal.pone.0129545 (PMC4511673; doi:10.1371/journal.pone.0129545)
Supplement: S1 Text — (DOCX) [file pone.0129545.s005.docx]

**Data availability**

The DOI for the dataset associated with this article is :

10.17037/DATA.1 (with the standard url of: [http://dx.doi.org/10.17037/DATA.1)](http://dx.doi.org/10.17037/DATA.1%29)

This leads to a holding entry on the LSHTM data repository (http://datacompass.lshtm.ac.uk/3/), which in turn provides a standard link to the ACT Consortium repository where all open access datasets for the ACT Consortium will be housed.  (This includes an optional registration page) Once CONTINUE option is selected it will take the user directly to the dataset and documentation associated with it. (The repository base address is <https://actc.lshtm.ac.uk> )
